# Health benefit/burden, PM_2_ _.5_ removal effectiveness, and power consumption based comparison of common residential air‐cleaning technologies in the United States

**DOI:** 10.1111/ina.13080

**Published:** 2022-07-19

**Authors:** Saloni Vijay, Jing Wang

**Affiliations:** ^1^ Institute of Environmental Engineering (IfU) Zürich Switzerland; ^2^ Laboratory for Advanced Analytical Technologies Dubendorf Switzerland; ^3^ Department of Mechanical and Process Engineering (MAVT) Global Health Engineering group Zürich Switzerland

**Keywords:** air cleaning technologies, air cleaning technologies comparison, disability‐adjusted life years, filter aging, indoor PM_2.5_, power consumption

## Abstract

This modeling study compared the common air cleaners in U.S. residences based on averted disability‐adjusted life years (DALYs) related to indoor PM_2.5_ concentration reduction and the DALYs resulted from carbon‐di‐oxide (CO_2_) emissions from power consumption. The technologies compared include mechanical fibrous filters, electret fibrous filters, and electronic air cleaners. For DALYs estimation, the indoor PM_2.5_ concentration and power consumption were first calculated and compared. These were then multiplied by the respective health damage factors. Air cleaners were compared under several indoor particle size distributions scenarios. A methodology was developed to evaluate the influence of the aging of air cleaners on the selected comparison criteria. The results suggest that the averted DALYs from indoor PM_2.5_ concentration reduction far supersedes the indirect DALYs associated with the operational power consumption of the air cleaners. Hence, the DALY‐based ranking of the air cleaners considered was the same as that of their effectiveness to reduce indoor PM_2.5_ concentrations. However, the result should be taken with care as only the use‐phase of air cleaners was considered. For future study, a complete life‐cycle assessment is recommended. Considering aging can change the ranking of the air cleaners and is thus advised to be incorporated in further studies.

**Practical Implications:**

- The results suggest important factors to be considered while comparing the air cleaning technologies for a particular indoor environment.
- The method developed allows comparing air cleaners without the burden‐shifting from indoor air pollution reduction by air cleaners to their power consumption.
- These associations persisted only for disinfecting wipes and were no longer observed for green and home‐made products, when considering the co‐use of irritants and sprayed products at home.

## 1 INTRODUCTION

The growing awareness about the ill‐effects of airborne particulate matter (PM) has encouraged the development of air‐cleaning technologies.^1^ Especially, PM_2.5_ (particles with an aerodynamic diameter less than 2.5 *μm*)—which can penetrate deep into the lungs, is known to cause adverse health impacts.^2^ Americans spend almost 58% of their time inside their home.^3^, ^4^ Occupants in residential buildings can be exposed to PM_2.5_ from both indoor and outdoor origin. Outdoor particulates can infiltrate through cracks in building envelope or through open windows.^5^, ^6^, ^7^ Also, some household activities like smoking and cooking can generate indoor particles.^8^, ^9^ Thus, air‐cleaning devices in indoor environments play an important role to clean air. However, at the same time, the operation of these devices consumes power.^10^ Power consumption leads to greenhouse gases emissions (GHGs) and is thus associated with adverse indirect health impacts.^11^ Overall, an ideal air purifier should be able to reduce indoor particles to safe levels with low power consumption and thus result in overall health benefits.

Different indoor air‐cleaning technologies are available in U.S. residential buildings. Broad categorization includes fibrous filters (FFs), electret fibrous filters (EFFs), and electronic air cleaners (EACs).^12^ The FFs are known to collect the dust particles from inflow air by mechanisms including diffusion, interception, and inertial impaction.^13^ In EFFs, the fibers carry an electric charge and, hence, additionally collect particles by electrostatic attraction.^14^ The commercial EAC usually consists of an electrostatic precipitator (ESP) with a pre‐filter mesh for removing large particles and a post‐filter for odor removal.^12^ In ESP, the particles are passed through a strong electric field and afterward collected on alternatively charged or grounded collection plates.^13^

All these air cleaners have their advantages and disadvantages. In general, FFs can achieve high filtration efficiency, but at the cost of high‐pressure drop which leads to large power consumption.^15^ Both filtration efficiency and pressure drop tend to increase with the accumulation of dust over the filter with usage/loading.^12^ New EFF can achieve higher filtration efficiency compared to the uncharged FF having the same filter parameters.^14^ It is due to the additional electrostatic attraction of particles toward the charged fibers. However, with loading, the charges can be shielded by the collected particles, which reduces the in‐use filtration efficiency initially. After a substantial build‐up of the dust cake, filtration efficiency starts increasing similar to other uncharged FFs.^12^ EACs generally have lower pressure drop compared to FFs and EFFs.^12^, ^15^ However, additional power is required to ionize the gas molecules. With use, EAC's filtration efficiency may decrease, and pressure drop may not change much.^12^

Several modeling studies have been done in the past to compare residential air cleaners in different scenarios.^6^, ^16^, ^17^, ^18^ The comparison was usually conducted based on minimum efficiency reporting value (MERV). El Orch et al^6^ evaluated the size‐resolved infiltration factors for U.S. residences in several scenarios, including usage of different MERV rating filters. Azimi et al^16^ compared the single‐pass PM_2.5_ and ultra‐fine particles removal efficiency of different MERV rating filters for particles of outdoor origin. Fisk et al^17^ compared the indoor particulate mass reduction, energy consumption, and operational cost of different in‐duct and stand‐alone filters. Riley et al^18^ examined the influence of different parameters over indoor PM concentrations, including filters. Waring and Siegel^19^ estimated particle loading rates of HVAC filters in different scenarios. The filtration efficiency was assumed to be the same as from new filters even after dust loading. These studies either neglected the impact of loading on filtration efficiency and pressure drop or ignored the power consumption of these technologies. Also, none of these studies focused on the health benefits/burden of using air cleaners.

Several studies tried to incorporate indoor air quality into life‐cycle assessment (LCA).^4^, ^20^, ^21^, ^22^ The main purpose of installing air cleaners in the house is to provide a healthy environment to individuals.^10^ However, the indirect health impacts associated with power consumption due to carbon‐di‐oxide (CO_2_) emissions are not estimated. 1 kg equivalent of CO_2_ emissions (customary100‐year global warming potential values) is associated with a damage factor of 2 to 6.2 *×* 10^−7^ Disability‐Adjusted Life Years (DALYs).^11^ As per the World Health Organization definition, one DALY represents the loss of the equivalent of one year of full health. Bragoszewska et al^22^ conducted an LCA case study of an air purifier and evaluated the human health endpoint category from indoor bio‐aerosols reduction, electricity consumption, and other factors. However, it is not very clear if the impact of indoor PM_2.5_ reduction was included.

The figure of merit (FOM), representing the ratio between filtration efficiency and pressure drop, is often used to compare air cleaners as higher FOM indicates better performance air cleaner.^23^ However, FOM may not be sufficient to evaluate the suitability of a device in a particular environment. Sources and strengths of residential indoor PM_2.5_ vary substantially from one indoor environment to another.^6^, ^19^ A high filtration efficiency device but having high‐pressure drop may be necessary for scenarios having high indoor PM_2.5_, to bring the concentration to a safe level. However, in some lower concentration scenarios, the same high filtration efficiency filter may not be desirable and would unnecessarily lead to high power consumption, and hence, may have an overall adverse health impact. For this, DALY offers more flexibility as it varies for different indoor PM_2.5_ concentration scenarios.

This study compares the averted DALYs due to a decrease in indoor PM_2.5_ concentration and DALYs increase resulting from power consumption among three residential air‐cleaning technologies commonly used in U.S. residences, namely EAC, EFF, and FF. For this, the study first estimated and compared indoor PM_2.5_ concentration and power consumption. The evaluation used different indoor PM_2.5_ sources and a range of air exchange rates, particle deposition loss, and penetration factors.

## 2 METHODOLOGY

### 2.1 Residential building model

**FIGURE 1:**
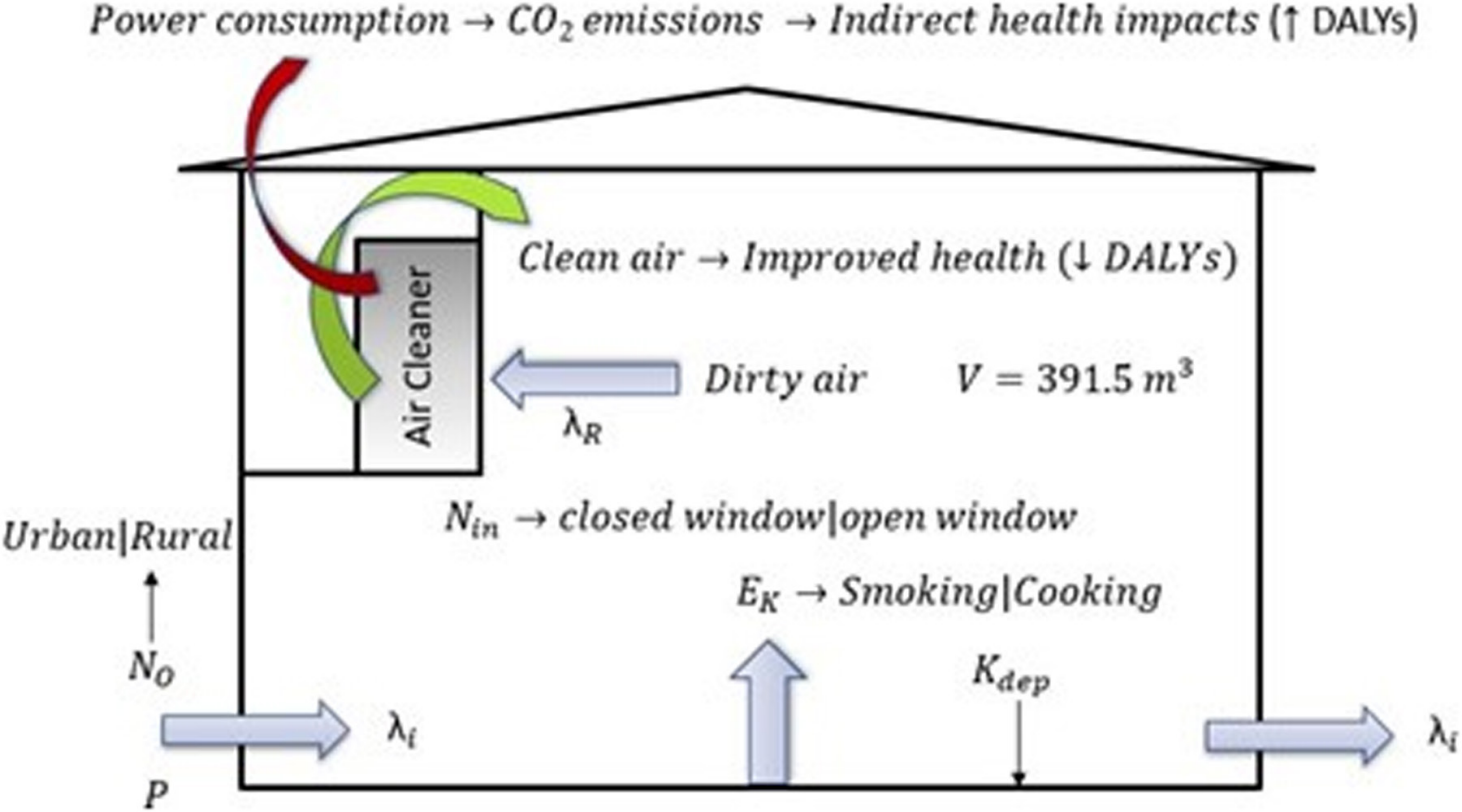
One box model of a residential building having the air‐cleaning device installed. It is representing the fate and transport of pollutants in the building envelope. The implication of clean air and power consumption by air cleaners is also indicated

A completely mixed one‐box model was assumed as a representative of residential building envelope (Figure 1). *N*_*in*_ and *N*_*o*_ were the indoor and outdoor size‐resolved particle number concentration distributions [#*/cm* ^3^], respectively; *E*_*k*_ was the particulate size‐resolved emission rate [#*/h*] from indoor source k; *V* was the room volume [*m* ^3^]; *λ*_*i*_ and *λ*_*R*_ represented the air exchange rate (AER) and airflow re‐circulation rate through the filter [1*/h*], respectively; *K*_*dep*_ was the size‐dependent particulate deposition loss rate on all surfaces of the residential envelope [1*/h*]; *P* represents the size‐resolved penetration factor of outdoor air in indoor environment [#].

Figure 1 was similar to the building model used by Riley et al,^18^ except for the absence of the building mechanical ventilation air intake system, as in most residential buildings, the fresh air enters only through natural ventilation via doors or windows, or through infiltration (cracks in building envelope).^19^ Major assumptions were—no concentration gradient near the source of indoor emission and no re‐suspension or coagulation of particles. The consideration of re‐suspension would be important for coarse particles.^18^

### 2.2 Data Collection

#### 2.2.1 Particle size distributions (PSDs)

Several indoor PM_2.5_ concentration scenarios were formed. Indoor emissions were assumed to be from either one or both cooking and smoking. The study included both urban and rural location PSDs. The scenarios and their explanation were provided in Table A.1 (SI A.1.1). The PSDs (ambient air in rural and urban locations, and those from indoor emission sources—cooking and smoking) were from Waring and Siegel.^19^ The parameters of all the PSDs (Table A.3) and further discussion were given in SI A.1.3.

#### 2.2.2 Penetration factor, deposition loss, air exchange, and re‐circulation rate

The entry of outdoor particles in indoor air was assumed under two situations—One where the particles enter only through infiltration, termed as “closed window scenario”; another when the particles enter from open doors and windows, referred to as “average open window scenario.” In the closed window scenario, the assumption was that windows and doors are closed all the time, and the particles can only enter from cracks in the building. The average open window scenario assumes the mild weather condition when 20% of the time, windows were open to a large extent (high window opening) and 80% of the time, to a low extent (low window opening).^6^ In total, there were 16 PSDs.

Air exchange rate (AER, *λ*_*i*_, 1*/h*), particle size‐resolved penetration factor (*P*), and size‐based deposition loss rate (*K*_*dep*_, 1*/h*) were obtained from El Orch et al^6^ for the closed window situation. The method suggested in the same study was used for the average open window case (SI A.1.3).

The geometric mean AER of 0.44 1*/h* in a closed window scenario was used.^6^ The AER in average open window scenario was calculated as 1.056 1*/h*, using Equation A.2 (SI A.1.3). The mean size‐resolved deposition loss rate in closed window scenario was taken as *K*_*dep*_ = 1.06 + 1.83*log*(*d*_*p*_) + 1.65*log*(*d*_*p*_)^2^ from Figure 3 (b) in El Orch et al.^6^ For average open window case, it was calculated as 1.324 times the *K*_*dep*_ using Equation A.3 (SI A.1.3).

A tri‐modal log‐normal distribution was fitted to the penetration factor curve for closed window case given in Figure 3 (a) of El Orch et al.^6^ The obtained distribution parameters can be found in Table A.4 (SI A.1.3). To obtain the distribution in average open window scenario, Equation A.4 (SI A.1.3) was used. The deposition loss rate and penetration factors were given for particles of size less than 10 *μm*. The same curves were extended for larger diameter particles as given in Figure A.1 (SI A.1.3).

The air re‐circulation rate (*λ*_*R*_, *h* ^*−*1^) across the air cleaner was calculated as *λ*_*R*_ = *Q*_*r*_ */V*, where *Q*_*r*_ was the airflow rate through the air cleaner [*m* ^3^ */h*] and *V* was the volume of the room [*m* ^3^]. The volume of the residence was from Waring and Siegel,^19^ that is, 391.9 *m* ^3^. The *Q*_*r*_ of 1391.5 *m* ^3^ */h* was used.^12^ The calculated *λ*_*r*_ was 3.55 *h* ^*−*1^, which was close to the *λ*_*r*_ of 4 *h* ^*−*1^ in the “residential” case of Riley et al.^18^

#### 2.2.3 Filtration efficiency and pressure drop

Hecker and Hofacre^12^ published the pressure drop data and size‐resolved filtration efficiency for commonly used air‐cleaning devices in U.S. residential buildings. The reported air‐cleaning devices include 2 FFs, 6 EFFs, and 3 EACs. The MERV rating and pressure drop of these devices (new device) were given in Table A.2 (SI A.1.3). Out of the 11 air cleaners, 5 were selected (Table 1). For rationale behind the selection, refer to SI A.1.2.

**TABLE 1:** Selected residential air cleaners for technology comparison. “PD” represents the pressure drop at flow rate of 1391.5 m^3^/h. “Original name” refers to the name used by Hecker and Hofacre^12^

Air cleaner name	Type	MERV Rating	PD [Pa]	Aging Data	Original name
FF6	Pleated fibrous filter	6	47	No	NS
EFF7	Pleated electret filter	7	35	Yes	DDUE
EFF12.1	Pleated electret filter	12	107	Yes	NM
EFF12.2	Pleated electret filter	12	22	No	FUA
EAC14	Electronic air cleaner	14/15	15	Yes	Unit P

The selected air cleaners were renamed using the type of technology followed by the MERV rating. For example, “NS” an FF with MERV 6 was renamed as FF6. As two EFFs (“NM” and “FUA”) have MERV rating 12, these were renamed as EFF12.1 and EFF12.2, respectively. Table 1 shows the pressure drop and the modified names. The filtration efficiency and pressure drop data were at the flow rate of 1391.5 *m* ^3^ */h*. The experimental filtration efficiency data in Hecker and Hofacre^12^ were given in Figure A.2 in SI A.1.3. The pressure drop data for aged device case were given in Table A.5 in SI A.1.3.

### 2.3 Indoor PM_2_ _.5_ concentration

To obtain indoor PM_2.5_ concentration, first the indoor size‐resolved particle number distribution *N*_*in*_ was calculated by Equation 1.
(1)

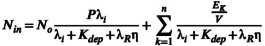

Outdoor particles can enter indoor depending upon penetration factor (*P*) and AER (*λ*_*i*_, 1*/h*). Emission from indoor source *k* can dilute due to the air exchange with outdoor air (*λ*_*i*_ *,* 1*/h*). Also, some indoor particles can deposit on the surfaces in the building. Particle deposition loss rate was represented by *K*_*dep*_ [1*/h*]. Air flow into the device with air re‐circulation rate of *λ*_*R*_ [1*/h*]. Particulates were removed from indoor air depending upon the device size‐resolved filtration efficiency (*η*). The experimental filtration efficiency data reported in Hecker and Hofacre^12^ were for particles of diameter between 0.03 and 10 *μm* (Figure A.2 in SI A.1.3). Continuous functions were obtained by curve‐fit, explained in SI A.1.4.

Equation 1 was also used by Waring and Siegel.^19^ It was a combination of two equations—the indoor fraction from the outdoor environment (original model by Riley et al^18^), and the particles originating indoors (used by Nazaroff and Klepeis^24^). The equation assumed that the parameters were constant in time and were uncorrelated with the indoor and outdoor PSDs.^19^

To get indoor PM_2.5_ concentration for new device case, the particle number distribution from Equation 1 was converted to mass distribution. The assumption was that all particles were spherical with a constant unit density. Afterward, the mass distribution was integrated numerically between the particle size of 0.001 to 2.5 *μm*, taking the bin size of 0.001 *μm*. Formula can be found in SI A.1.5 (Equation A.13).

For the aged device case, PM_2.5_ concentration was calculated as the time‐weighted average of three months. Time period of three months was taken as it was the recommended life of air cleaners tested by Hecker and Hofacre.^12^ Also, the data of filtration efficiency and pressure drop collected by Hecker and Hofacre^12^ were for the span of three months. In the case of aged EFFs, the mass deposition over the filters with time was available. The data of EFF7 were available at subsequent loading of 1, 8, 7, and 5 g. For EFF12.1, the filtration efficiency curves were available at subsequent PM mass deposition of 2, 1, 3, and 9 g. The assumption was that the filtration efficiency does not change between the successive loading intervals. The mass deposited on the filter (*m*_*f*_ *, g*) in 1 day was calculated by integrating Equation 2 over the bin size of 0.001 *μm* for the entire indoor particle size range.

(2) $$m_{d}=N_{m}\times \eta \times Q_{r}\times 24\times 10^{-6}\;$$

where *m*_*d*_ was the size‐resolved particle mass deposition over the filter [g]; *N*_*m*_ was the size‐resolved indoor particle mass distribution [*μg/m* ^3^] (Equation A.1, SI A.1.3); *η* was the size‐resolved filtration efficiency of air‐cleaning device; *Q*_*r*_ was the air re‐circulation rate taken as 1395.1 *m* ^3^ */h*. The factor 24 was to convert hour to days and factor 10^−6^ converts the *μg* to *g*. Hence, the total time in days (*t*_*m*_) to deposit z *g* mass over the filter was calculated as *t*_*m*_ = *z/m*_*f*_.

For EFFs, the time to accumulate subsequent masses depended upon the indoor air particulate concentration. Thus, it varied from one scenario to another. In the case of a high indoor PM concentration scenario, the PM mass should deposit faster over the filter, resulting in faster aging. The indoor PM_2.5_ concentration was calculated at all the filtration efficiency curves (available at different loading—new and aged device). The time‐weighted average of indoor PM_2.5_ concentration was taken by using the times calculated for depositing a particular loaded mass.

For aged EAC, the PM_2.5_ concentration was calculated at different filtration efficiency curves in time, and then, the time‐weighted average was taken. The recommended life of the EFFs used in this study was three months. Although the EAC life was longer, for a reasonable comparison, the same period was used. Elaborated method for the aged devices indoor PM_2.5_ concentration calculation was given in SI A.1.5.

The above steps were repeated assuming size‐resolved densities in place of constant unit density. This was to check whether the ranking of performance of air cleaners was affected by the density assumption or not. For particles of size lower than 10 *μm*, same size‐resolved densities were used as that used by Azimi et al^16^ (Table 2). For particles of size larger than 10 *μm*, density of 2.5 *g/cm* ^3^ was assumed.^25^

**TABLE 2:** Size‐resolved particle densities used for particles of size less than 10 μm

Particle diameter (*d*_*p*_)	Density [g/cm^3^]
*d*_*p*_ <0.14 μm	1.3
0.14 μm ≤ *d*_*p*_ <0.42 μm	1.4
0.42 μm ≤ *d*_*p*_ <1.2 μm	1.5
1.2 μm ≤ *d*_*p*_ <3.5 μm	1.6
3.5 μm ≤ *d*_*p*_ <10 μm	1.7

### 2.4 Power consumption

For FF and EFF, during operational phase, power consumption was only to overcome pressure drop, termed as fan power (*P*_*f*_, *W*). It was calculated as *P*_*f*_ = *Q*_*r*_∆*p*_*f*_ */*(*η*_*fan*_.3600), where *Q*_*r*_ was the air flow rate through the air‐cleaning device in *m* ^3^ */h*, ∆*p*_*f*_ was the pressure drop experienced by air‐cleaning device [*Pa*], and *η*_*fan*_ was the fan efficiency. The factor 3600 was to convert time in *h* to *s*. *η*_*fan*_ was taken as 0.5 in this study and *Q*_*r*_ was the same as mentioned in Section 2.2.3, that is, 1391.5 *m* ^3^ */h*.

In the case of EAC, additional power was required to charge the particles, termed as device power (*P*_*device*_, *W*) calculated as *P*_*device*_ = *V*_*o*_ *I*, *V*_*o*_ was the voltage of the electrodes [*V*], taken as 6.2 *kV* and *I* was the discharge current [*A*] taken as 2.5 *mA*. The EAC device power calculated was compared with values from other literature. The total power consumption by EAC was the sum of the device power and fan power (*P*_*eff*_ = *P*_*device*_ + *P*_*f*_). The fan power calculation for EAC was the same as that for FF and EFF. The power consumption of an aged device also depended upon the loading. Hence, time‐weighted power consumption was calculated using the times calculated in Section 2.3.

### 2.5 DALYs from indoor PM_2_ _.5_

The DALYs associated with indoor PM_2.5_ concentration were calculated by Equation 3.

(3) $${\text{DALY}}_{\mathrm{PM}2.5}={\mathrm{EF}}_{\mathrm{PM}2.5\to \text{DALY}}\times C_{\mathrm{PM}2.5}\times B\times N\times 10^{-9}\times t_{r}$$

where *EF*_*PM*2.5 *→ DALY*_ was the effect factor that calculates the DALYs associated with PM_2.5_ inhalation [*DALY s/kgPM*_2.5_ *inhaled*], *C*_*PM*2.5_ was the PM_2.5_ concentration in indoor environment [*μg/m* ^3^], B was the volume of air intake by one person in one year [*m* ^3^ */yr/person*] (also called inhalation rate, IR), N was the number of persons in the residence, and *t*_*r*_ was the total time‐span considered, that is, 1 year. The factor 10^*−*9^ was to convert *C*_*PM2.5*_ from *μg/m* ^3^ to *kg/m* ^3^.

Equation 3 was derived from the study by Rosenbaum et al.^4^ A general formula for determining DALYs associated with indoor emissions [*DALY s/kgPM*_2.5*emitted*_] was simplified, that is, *CF*_*PM*2.5*emissions → DALY*_ = *EF.IF*, where *CF*_*PM*2.5*emissions → DALY*_ was the characterization factor to calculate DALYs due to PM_2.5_ emissions. IF was the PM_2.5_ intake fraction calculated as $\text{IF}=\frac{B.N}{V.\mathrm{Kex}}$, where B was the volume of air intake by one person in one year [*m* ^3^ */yr/person*], *K*_*ex*_ was the overall ventilation rate in the indoor environment [*h* ^*−*1^].^4^ The indoor PM_2.5_ emission rate divided by the *K*_*ex*_, and room's volume *V* was the PM_2.5_ concentration in the room (Equation 1 and SI Equation A.13).

*EF*_*PM*2.5 *→ DALY*_ was taken as 78 to 110 *DALY/kgPM*_2.5_ *inhaled*, from the study by Gronlund et al^26^ (detail about *EF*_*PM*2.5 *→ DALY*_ in SI A.1.7). The value of B was taken from the same study as 4745 *m* ^3^ */yr/person*. The number of persons in one residence was assumed as 2.6 *persons/residence*.^4^

### 2.6 DALYs from power consumption

Equation 4 was used to calculate the DALYs associated with the power consumption of air cleaners.

(4) $${\text{DALY}}_{P}=\frac{{\mathrm{CF}}_{\mathrm{CO}2\to \text{DALY}}\times C_{\text{electricity}\to \mathrm{CO}2}\times P_{\mathrm{eff}}\times t_{r}}{1000}$$

where *DALY*_*P*_ was the DALYs associated with total power consumption, *CF*_*CO*2 *→ DALY*_ was the DALY characterization factor [*DALY/kgCO*_2_], *C*_*electricity → CO*2_ was the conversion factor to convert electricity consumption to equivalent CO_2_ emissions [*kgCO*_2_ */kWh*], *P*_*eff*_ is the effective power consumption [*W*] of air‐cleaning device, and *t*_*r*_ was the device run time in hours. Division by 1000 was to convert *P*_*eff*_ from *W* to *kW*.

As per the U.S. energy information administration, in 2019, 0.427 kg CO_2_ was emitted per kWh of electricity consumption in the United States.^27^ This value was used for *C*_*electricity → CO*2_. Tang et al^28^ calculated that 1 kg CO_2_ emissions (customary 100‐year global warming potential values) are associated with damage factor of 2 to 6.2 *×* 10^−7^ DALYs. This range was used for *CF*_*CO2 → DALY*_ (details in SI A.1.7). The device was assumed to run 24 h each day in a year. Both *DALY*_*P*_ and *DALY*_*PM*2.5_ were the DALYs from air‐cleaning device installed in 1 residence.

## 3 RESULTS AND DISCUSSION

### 3.1 Indoor PM_2_ _.5_ concentration

**FIGURE 2:**
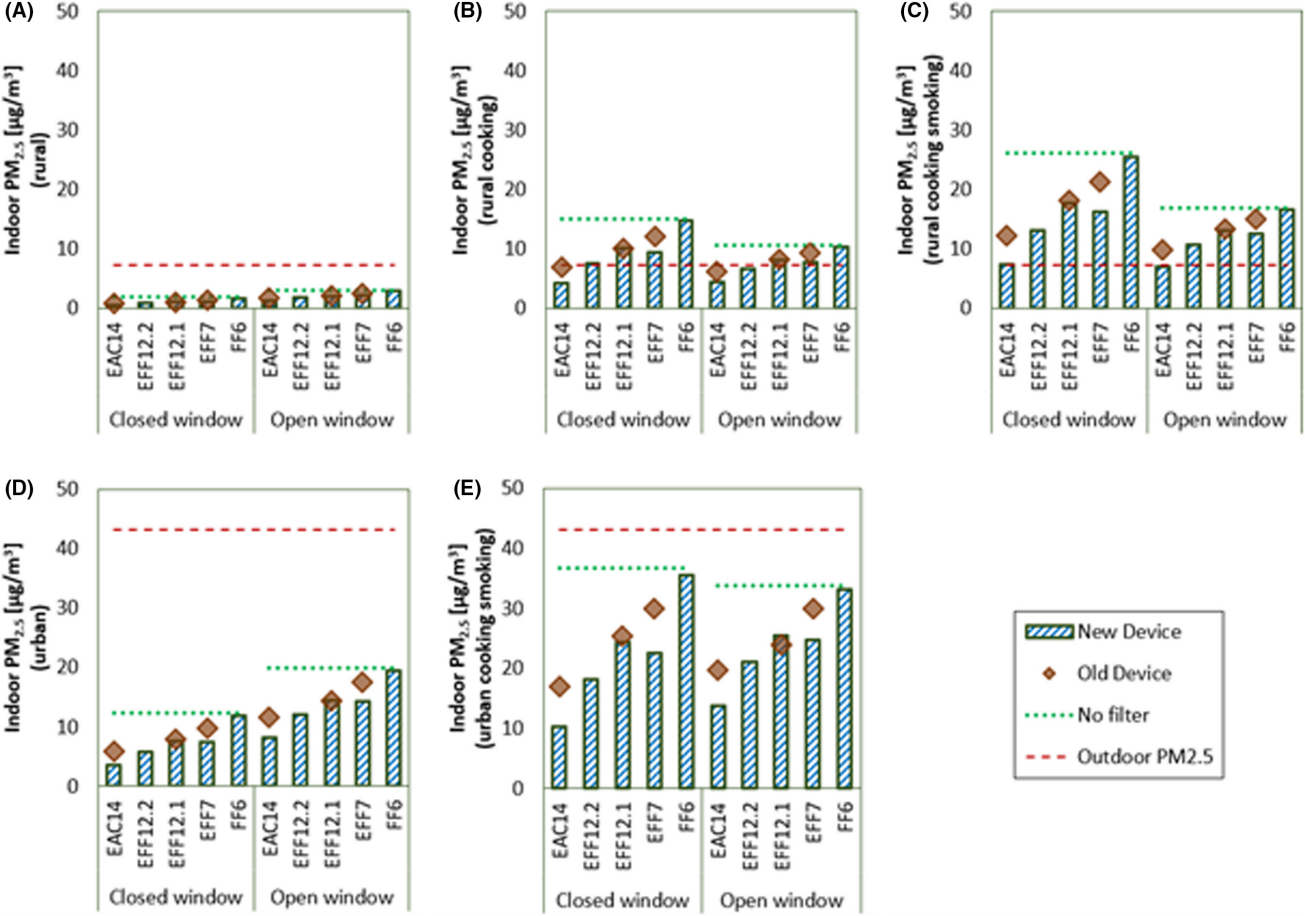
Indoor PM_2.5_ concentration with constant density assumption in—(A) Rural, (B) Rural cooking, (C) Rural cooking smoking, (D) Urban, and (E) Urban cooking smoking scenario. For all the scenarios, both closed and average open window situations are shown. Comparison of different air‐cleaning technologies is made. Lower concentration implies better performance

Comparison of air‐cleaning technologies in terms of indoor PM_2.5_ concentration in different scenarios under constant unit density assumption is shown in Figure 2. The results for rural smoking and urban smoking scenario are given in SI A.2.3 (Figure A.7), as the resulting PM_2.5_ concentration was very close to rural cooking and urban cooking scenario, respectively. This can be because the 24‐hour‐averaged PM emission rates were approximately the same in both the cases (5.8 and 5 *mg/h* in case of cooking and smoking, respectively—refer SI A.1.3).

A higher concentration of indoor PM_2.5_ implies lower effectiveness of the air cleaner. In all the scenarios, for the new device case, FF6 is the least effective, and EAC14 is most effective in reducing indoor PM_2.5_ concentration. Among the EFF12.1 and EFF12.2, EFF12.2 is more effective. Surprisingly, EFF7 performance is better than EFF12.1, despite having a significantly lower MERV rating of 7. The reason is, for particles of size lower than 0.3 *μm*, the size‐resolved filtration efficiency of EFF7 is higher than that of EFF12.1 (Figure A.6 (a) in SI A.2.2). Note that the MERV rating is based only on the filtration efficiency for particles of sizes greater than 0.3 *μm*.^16^ Hence, the results suggest the need to incorporate the filtration efficiency of lower diameter particles while rating the commercial filters. Same is stated by Hecker and Hofacre,^12^ and Azimi et al.^16^ The results are somewhat biased as only very low MERV FF was taken for comparison. However, Hecker and Hofacre^12^ reported that only the low MERV FFs are common in U.S. residences.

For the aged device case, as represented by translucent brown diamonds in Figure 2, the PM_2.5_ removal effectiveness is again highest in case of EAC14. This is even after selecting the worst filtration efficiency EAC among the three EACs reported in Hecker and Hofacre^12^ (details in SI A.1.2). However, among EFF12.1 and EFF7, the ranking is reversed compared to new filters. The overall effectiveness of aged EFF7 is lower than that of EFF12.1. Thus, only considering the filtration efficiency of new filters is insufficient to judge the air cleaner in a real‐life situation.

The indoor PM_2.5_ concentration after changing the unit density assumption to that mentioned in Sec. 2.3 is given in Figure A.8 and Figure A.9 in SI A.2.3. The density assumption does not affect the relative ranking of new and aged air cleaners. More discussion is given in SI A.2.3. The method developed for aged device cases can be replicated in similar modeling studies, provided the filtration efficiency after certain mass depositions are known.

### 3.2 Power consumption

**FIGURE 3:**
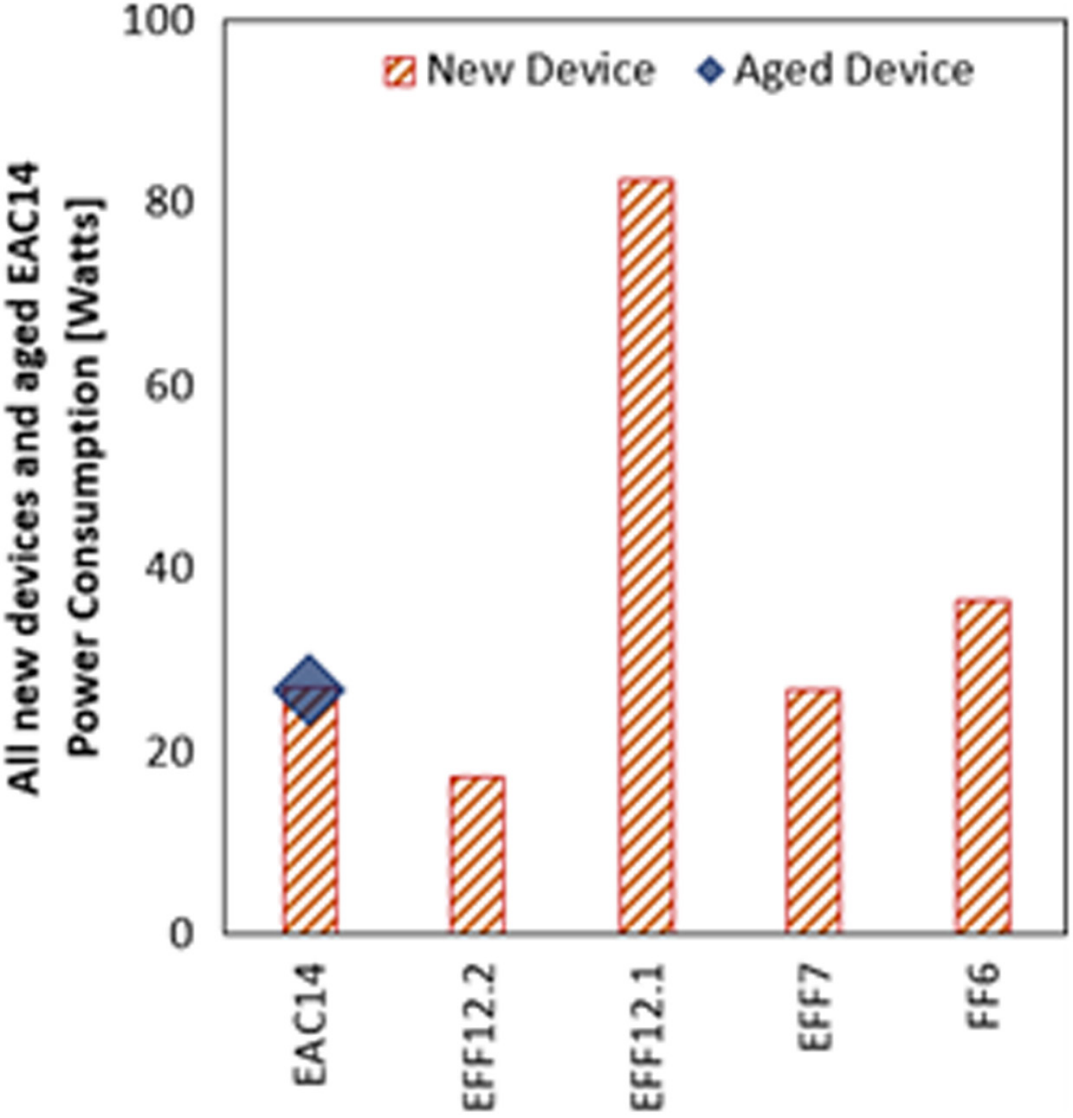
Effective power consumption of all new devices and aged EAC14

The power consumption of all new devices and aged EAC14 is given in Figure 3. The power consumption of new filters is independent of the indoor PM concentration. The power consumption of EFF12.2 is the lowest attributed to its low pressure drop, followed by EAC14. The pressure drop of EAC14 is the lowest; however, apart from the fan power, additional power is consumed. The EAC device power is 15.5 *Watts*, which is near to the value obtained by Blondeau et al^29^ of 15 *Watts* for a commercial ESP as measured by Wattmeter. Among the three EFFs and FF6, the ranking of power consumption is the same as that of the pressure drop, which makes sense as the same conversion factor is multiplied by all. Overall, for the new devices, no clear conclusion can be made that a particular technology is less power‐consuming than others.

**FIGURE 4:**
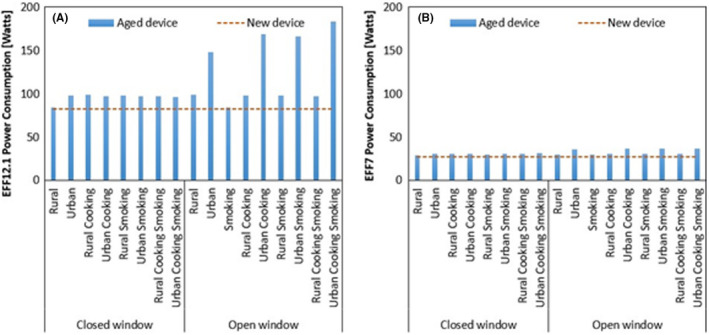
Effective power consumption of—(A) EFF12.1 and (B) EFF7 aged device shown by blue bars compared to respective new device power consumption shown by red dotted line

The power consumption of the aged EFF12.1 and EFF7 is shown in Figure 4 (a) and (b), respectively. It can be seen that the power consumption of aged devices is higher than that of the new device. This increase is highly dependent upon the indoor PM concentration. The higher the indoor PM concentration, the faster the mass would deposit over the filter. It leads to higher power consumption in the case of aged devices. Among EFF12.1 and EFF7, a higher increase is observed for EFF12.1, as its aging rate is faster. It means that the time for filtration efficiency to become higher than that of the new filter is shorter. The analysis shows that considering only the new filter power consumption can be misleading. Because of aging, the power consumption of EFF can even double.

The power consumption of new devices was independent of the density assumption. However, as pressure drop is affected by mass deposition over the filter, the power consumption of aged EFF7 and EFF12.1 was affected. The aged EFF12.1 power consumption was 34% higher with the size‐dependent density assumption than the unit density assumption, in the case of the fastest loading scenario, that is, urban cooking smoking. In the case of EFF7, it increased by 13% in the same scenario, but the relative ranking was still not affected by the density assumption (Figure A.10 in SI A.2.4).

### 3.3 Disability‐adjusted life years

Simplifying Equation 3 in Sec. 2.5 results in 9.62 *×* 10^*−*4^ to 13.6 *×* 10^*−*4^ DALYs reduction per residence for 1 *μg/m* ^3^ of decrease in PM_2.5_ concentration after the installation of air‐cleaning device. A precaution should be taken while using the effect factor from Gronlund et al^26^ of 78 to 110 DALY per kg PM_2.5_ inhaled. This factor should only be used when the indoor concentrations are comparable to outdoors. In case of very high indoor concentrations, a non‐linear concentration‐response function is recommended.^30^

From Equation 4 (Section 2.6), it is derived that 1 Watt extra power consumption in one residence may lead to 7.5 *×* 10^*−*7^ to 23.2 *×* 10^*−*7^ additional DALYs. It is by assuming full‐time usage of the device throughout the year. Notably, only the DALYs associated with CO_2_ emissions are considered. There are other GHGs emitted from the U.S. electricity generation, like CH_4_, N_2_O, and SF_6_, but the amount is smaller compared to CO_2_ emissions.

The DALY value associated with 1 *μg/m* ^3^ change in PM_2.5_ concentration is approximately 1000 times more than that with 1 Watt power consumption. With unit density assumption, the absolute PM_2.5_ reduction in different scenarios after installation of air cleaner ranges between 0.12 and 26 *μg/m* ^3^ (Sec. 3.1 and SI A.1.5), whereas, the power consumption ranges between 17 to 183 Watts (Section 3.2). In no situation, the health benefit through PM_2.5_ reduction is lesser than the health burden related to CO_2_ emission from power consumption. Thus, the ranking based on the DALY criterion is similar to what is shown in Section 3.1. A common way to evaluate the air cleaner performance is by the figure of merit (FOM). FOM‐based comparison is given in SI A.2.5.

The energy consumption during the manufacturing and the end‐of‐life treatment phase of air cleaners is not considered in this study. The FF and EFF have the recommended life of only three months. An LCA study by Kiamili et al^31^ concluded that the filters might have a significant contribution to the building life‐cycle energy consumption, attributed to their short life (total filter production phase impacts are high). A case study by Bragoszewska et al^22^ reported that the health damage points from electricity consumption exceed the health improvement from a purified air by an air cleaner (points are the relative weighting assigned to different damage categories). This study was in Poland, where the major source of electricity is coal. DALY from electricity consumption may vary depending upon the country's electricity mix. Nonetheless, using only the operational phase of air cleaners to quantify the DALYs can be misleading. Hence, a complete life‐cycle‐based including the end‐of‐life treatment phase DALYs calculation is required. Also, the impact of bi‐products from air‐cleaning technologies should be included. However, this is out of the scope of this study. The current study should not be mistaken with a cradle to cradle LCA. It does not follow the standard conventions of LCA. A very simplified calculation is done—that multiplies the respective health damage factors to PM_2.5_ concentration and power consumption to evaluate the relative importance of their respective DALYs increase/decrease for the use‐phase of air cleaners. However, it paves the way for LCA‐based DALYs comparison of air‐cleaning technologies. Notably, the method for accounting aging while calculating the indoor PM_2.5_ and power consumption was developed, which can be used in LCA studies. Also, scenario‐specific calculations are shown. Finally, combining indoor air quality with the energy consumption of devices in LCA to evaluate the air cleaners is recommended.

## 4 CONCLUSION

The criterion DALY was used to measure direct and indirect health benefits/burdens from air cleaners. This study revealed that the reduction in indoor PM_2.5_ concentration improved the DALYs far more than the indirect DALYs associated with the operational power consumption of the air cleaners in United States. However, the results should be taken with care as only the use‐phase was considered. Future research considering the entire life‐cycle including end‐of‐life treatment phase of the air cleaners is needed.

For new devices (constant filtration efficiency and pressure drop over time) and aged devices (changing filtration efficiency and pressure drop over time), the effectiveness to remove indoor PM_2.5_ was the best for EAC14 followed by EFFs. No clear deduction could be made for power consumption.

It was observed that the aging of a filter can change the ranking of the air cleaners. Aged EFF7 removed PM_2.5_ more effectively than aged EFF12.1. The results were the opposite for the new device case. Hence, it is recommended for modeling studies to consider the aging effect.

The current MERV rating is assigned by considering filtration efficiency for particles of diameter greater than 0.3 *μm*. It was observed that air cleaners with a higher MERV rating may not necessarily be more effective in reducing indoor PM_2.5_ compared to lower MERV rating cleaners. Thus, it would be recommended to adjust the MERV rating to include the filtration efficiency for particles of size less than 0.3 *μm*.

The study did not consider the harmful byproducts from the air cleaners. ESP technology is known for ozone emissions. Thus, the extension of this study should include the impact of byproducts in DALY calculation.

The study considers only the air cleaners that are commonly used in U.S. residential buildings. Thus, the evaluated performance ranking of the cleaners in this study is not a given. For office buildings, where high‐efficiency particulate absorbing (HEPA) filters are common, the ranking can be different. However, the methodology developed in this study is generalizable and can be extended to other situations.

## AUTHOR CONTRIBUTIONS

Not applicable, as number of authors are only two.

## FUNDING INFORMATION

The work was partially supported by Center for Filtration Research at University of Minnesota with the Subaward No. W530672710.

## CONFLICT OF INTEREST

No, there is no conflict of interest.

## PATIENT CONSENT STATEMENT

Not applicable.

## PERMISSION TO REPRODUCE MATERIAL FROM OTHER SOURCES

Not applicable.

## Supporting information


Appendix S1

## Data Availability

All data generated or analyzed during this study are included in this published article (and its supplementary information files).
