# Supplementary material for: Health benefit/burden, PM2 .5 removal effectiveness, and power consumption based comparison of common residential air‐cleaning technologies in the United States
Source: Indoor Air. 2022 Jul 19;32(7):e13080. doi: 10.1111/ina.13080 (PMC9543307; doi:10.1111/ina.13080)
Supplement: Supplementary file 1 — Appendix S1 [file INA-32-0-s001.docx]

# Supporting Information

## Methods - Air cleaners comparison

### Residential Building Model

Table A.1: Explanation of scenarios considered in this study

| Scenario | Explanation |
| --- | --- |
| Urban | Building located in Urban region; no source of indoor emission |
| Rural | Building located in Rural region; no source of indoor emission |
| Urban cooking | Building located in Urban region; indoor emissions only from cooking |
| Rural cooking | Building located in Rural region; indoor emissions only from cooking |
| Urban smoking | Building located in Urban region; indoor emissions only from smoking |
| Rural smoking | Building located in Rural region; indoor emissions only from smoking |
| Urban cooking smoking | Building located in Urban region; indoor emissions from both cooking and smoking |
| Rural cooking smoking | Building located in Rural region; indoor emissions from both cooking and smoking |

### Residential air cleaners selection

In this section, the rationale behind the selection of 5 air cleaners out of 11 is discussed. All the residential air cleaners commonly used in U.S. residences are summarized in [Table A.2,](#_bookmark1) as taken from Hecker and Hofacre.^1^ One important aspect during the selection of filters is their representativeness of the particular filter technology. Also, for old device case, it was important to have the change in filtration efficiency (FE) and pressure drop (PD) data of filters with time. No FF filter was tested for old device case by Hecker and Hofacre.[^1^](#_bookmark39) Thus, FF was used only for the comparison amongst new devices. Out of the two FFs, “NS” was selected for final comparison due to it’s higher MERV rating but almost similar PD as “PP” (T[able A.2).](#_bookmark1) Hecker and Hofacre[^1^](#_bookmark39) mentioned that they could not find any higher MERV rating FF amongst the commonly used devices in U.S. residences. The other cleaners using EEF or EAC technology were of MERV rating higher than or equal to 7. Thus, the selection of “NS” makes more sense to make the comparison process at least slightly fair. Both EFF’s for which the performance data during aging was available were selected, namely, “DDUE” and “NM”. These are used in both new and old devices comparison. From other electret filters, for new device comparison, FUA was also selected due to very high MERV rating and low PD. Because of the wide range of MERV rating and PD amongst the commonly used EFFs, none of these alone can be said to be a representative EFF. However, comparing them all can give a possible range of performance of EFF technology.

For EAC, aging data was available for all three of them. The FE of all three was almost similar and lies in MERV 14 and 15. For old device case, the FE of “Unit A” and “Unit H” do not vary significantly with time.[^1^](#_bookmark39) On the other hand, FE of Unit P decreased significantly [(Figure A.2](#_bookmark7) (b)). Thus, in order to evaluate the worst-case scenario for old device case of EAC, Unit P was selected for final comparison. The thought was to check if the worst performing aged EAC is better or worse than other EFF’s.

Table A.2: Residential air cleaners in report by Hecker and Hofacre^1^

| **Original name** | **Type** | **MERV Rating** | **PD* [Pa]** | **Aging Data** | **Selected for comparison** | **Air cleaning device name** |
| --- | --- | --- | --- | --- | --- | --- |
| PP | Pleated fibrous filter | 5 | 45 | No | No | - |
| NS | Pleated fibrous filter | 6 | 47 | No | Yes | FF6 |
| PAF | Pleated electret filter | 8 | 45 | No | No | - |
| RM | Pleated electret filter | 7 | 62 | No | No | - |
| AST | Pleated electret filter | 7 | 72 | No | No | - |
| DDUE | Pleated electret filter | 7 | 35 | Yes | Yes | EFF7 |
| NM | Pleated electret filter | 12 | 107 | Yes | Yes | EFF12.1 |
| FUA | Pleated electret filter | 12 | 22 | Yes | Yes | EFF12.2 |
| Unit P | Electronic air cleaner | 14 | 15 | Yes | Yes | EAC14 |
| Unit H | Electronic air cleaner | 15 | 29 | Yes | No | - |
| Unit A | Electronic air cleaner | 14 | 29 | Yes | No | - |

### Data Collection Particle size distributions

The PSDs is given in [Table A.3.](#_bookmark3) Ambient air PSDs, *N_o_* [#*/cm*^3^], were given as two tri-modal log normal distribution as a representative of urban, and rural region PSD, originally taken from Jaenicke[.](#_bookmark40)^2^ Same PSDs were used by Riley et al[^3^.](#_bookmark41) To check the plausibility of these distributions, the number distribution *N_o_* [#*/cm*^3^] was converted to mass distribution *N_m_* [*µg/m*^3^] by using [Equation A.1](#_bookmark4) given in [A.1.3.](#_bookmark2)

Afterwards, the integrated PM_2.5_, PM_10_ and PM_100_ levels in ambient air were calculated. For simplicity, the particle density of 1 *g/cm*^3^ was used and the shape was assumed to be spherical. The same assumption was used in other literature.[^3,^](#_bookmark41) [^4^](#_bookmark42) The ambient PM_2.5_, PM_10_ and PM_100_ concentrations for urban and rural region were obtained as 43, 60 and 91 *µg/m*^3^, and 7.3, 15 and 23 *µg/m*^3^, respectively. It should be noted that the PM_10_ and PM_100_ concentration is different from Waring and Siegel as their study assumed a density of 2.5 *g/cm*^3^ for particles of diameter greater than 2.5 *µm*. Also, note that the urban PM concentration may not be a representative for average U.S., where the mean annual PM_2.5_ was 8 *µg/m*^3^ in 2020, as reported by United States Environmental Protection Agency[^5^;](#_bookmark43) while the rural ambient PSD seemed okay. Still, the urban PSD was used as it can assess the suitability of air cleaners when the residence is located in highly polluted region.

A unimodal lognormal distribution was provided for size-resolved cooking emission rate. The distribution parameters were originally obtained by curve-fit on the mean cooking emissions strength data reported by Wallace et al[^6^.](#_bookmark44) The data were collected for 33 cooking episodes including frying on gas stoves[.](#_bookmark44)^6^ In the cooking scenario, 90 min of the cooking episode was assumed in 24 hours. Thus, to get daily average emissions, the episodic emission rate obtained from parameters given in [Table A.3](#_bookmark3) ( [A.1.3)](#_bookmark2) is multiplied by a factor of 0.0625. The same was done by Waring and Siegel[^7^.](#_bookmark45) The number distribution was converted to mass distribution by the same method as mentioned for ambient PSDs. The 24-hour average PM emission rate from cooking was obtained as 5.8 *mg/h*.

PSD for the smoking scenario was originally taken from Klepeis et al[^8^.](#_bookmark46) Mean smoking time of 6.5 min was assumed. For scenarios involving smoking emissions, the current study assumes that 14 cigarettes are smoked every day[.](#_bookmark47)^9^ Thus, the factor of 0.0632 was multiplied by the emission rate obtained from the parameters in [Table A.3](#_bookmark3). The daily average smoking emission rate was calculated to be 5 *mg/h*.

Table A.3: Parameters of particle size distribution for different scenarios. The table is taken from Waring and Siegel[^7^](#_bookmark45)

|  |  |  | Mode 1 | | | Mode 2 | | | Mode 3 | | |  |
| --- | --- | --- | --- | --- | --- | --- | --- | --- | --- | --- | --- | --- |
| Parameter | Distribution | Units | Total | Mean | Log10(SD) | Total | Mean | Log10(SD) | Total | Mean | Log10(SD) | Original Source |
| Ambient | Rural | (number/cm^3^) | 6650 | 0.015 | 0.225 | 147 | 0.054 | 0.557 | 1990 | 0.084 | 0.266 | Jaenicke^2^ |
|  | Urban | (number/cm^3^) | 99300 | 0.013 | 0.245 | 1100 | 0.014 | 0.666 | 36’400 | 0.05 | 0.337 | Jaenicke^2^ |
| Emission | Cigarette | (mg/h) | 79.2 | 0.2 | 0.322 | - | - | - | - | - | - | Klepeis et al^8^ |
|  | Cooking | (number/h) | 1.15E+14 | 0.06 | 0.287 | - | - | - | - | - | - | Wallace et al^6^ |

Outdoor particle number distribution is converted to mass distribution by [Equation A.1:](#_bookmark4)

$$Nm=Nn \rho_{p}\frac{{\pi d}_{p}^{3}}{6}$$

(A.1)

Where, *N_m_* is the particle mass distribution [*µg/m*^3^], *N_n_* is the particle number distribution [#*/cm*^3^], *ρ_p_* is the particle density, *d_p_* is the particle diameter.

### Penetration Deposition factors

Following equation from El Orch et al[^10^](#_bookmark48) was used to derive air infiltration exchange rate for open window scenario.

$$\text{λ}_{i, open}=\text{λ}_{i, closed}m_{low opening}f_{low opening}+\text{λ}_{i, closed}m_{high opening}\text{ }f_{high opening}$$

(A.2)

where, *λ_i,open_* is the air infiltration exchange rate for open window scenario [1*/h*], *λ_i,closed_* is the air infiltration exchange rate for closed window scenario [1*/h*], *m_low opening_* is the multiplication factor for low window opening (taken as 2), *m_high opening_* is the multiplication factor for low window opening (taken as 4), *f_low opening_* is the fraction of time window is open to low window opening (80%), and *f_high opening_* is the fraction of time window is open to high window opening (20%).

Following equation from El Orch et al[^10^](#_bookmark48) was used to derive size resolved particle deposition rate for open window scenario.

$$\text{K}_{dep, open}=\text{K}_{dep, closed}\alpha f_{low opening}+\text{K}_{dep, closed}\beta f_{high opening}$$

(A.3)

where, *K_dep,open_* is the particle deposition loss rate for open window scenario [1*/h*], *K_dep,closed_* is the particle deposition loss rate for closed window scenario [1*/h*], *α* is the multiplication factor for low window opening (taken as 1.7), *β* is the multiplication factor for low window opening (taken as 1.23), *f_low opening_* is the fraction of time window is open to low window opening (80%), and *f_high opening_* is the fraction of time window is open to high window opening (20%).

Following equation from El Orch et al[^10^](#_bookmark48) was used to derive size resolved penetration factors for open window scenario.

$$\text{P}_{open}=\text{(P}_{closed}\frac{\text{λ}_{closed}}{\text{λ}_{low opening}}+1\frac{\text{λ}_{low opening -}\text{λ}_{closed}}{\text{λ}_{low opening}}{)f}_{low opening}+1f_{high opening}$$

(A.4)

where, *P_open_* is the size-resolved particle penetration factor for open window scenario [#], *P_closed_* is the size-resolved particle penetration factor for closed window scenario [#], *λ_closed_* is the air exchange rate for closed window scenario, *λ_low opening_* is the air exchange rate for low window opening, *f_low opening_* is the fraction of time window is open to low window opening (80%), and *f_high opening_* is the fraction of time window is open to high window opening (20%).

The size-resolved penetration factor and particle deposition rate curves for open and closed window scenario is given in [Figure A.1.](#_bookmark5)


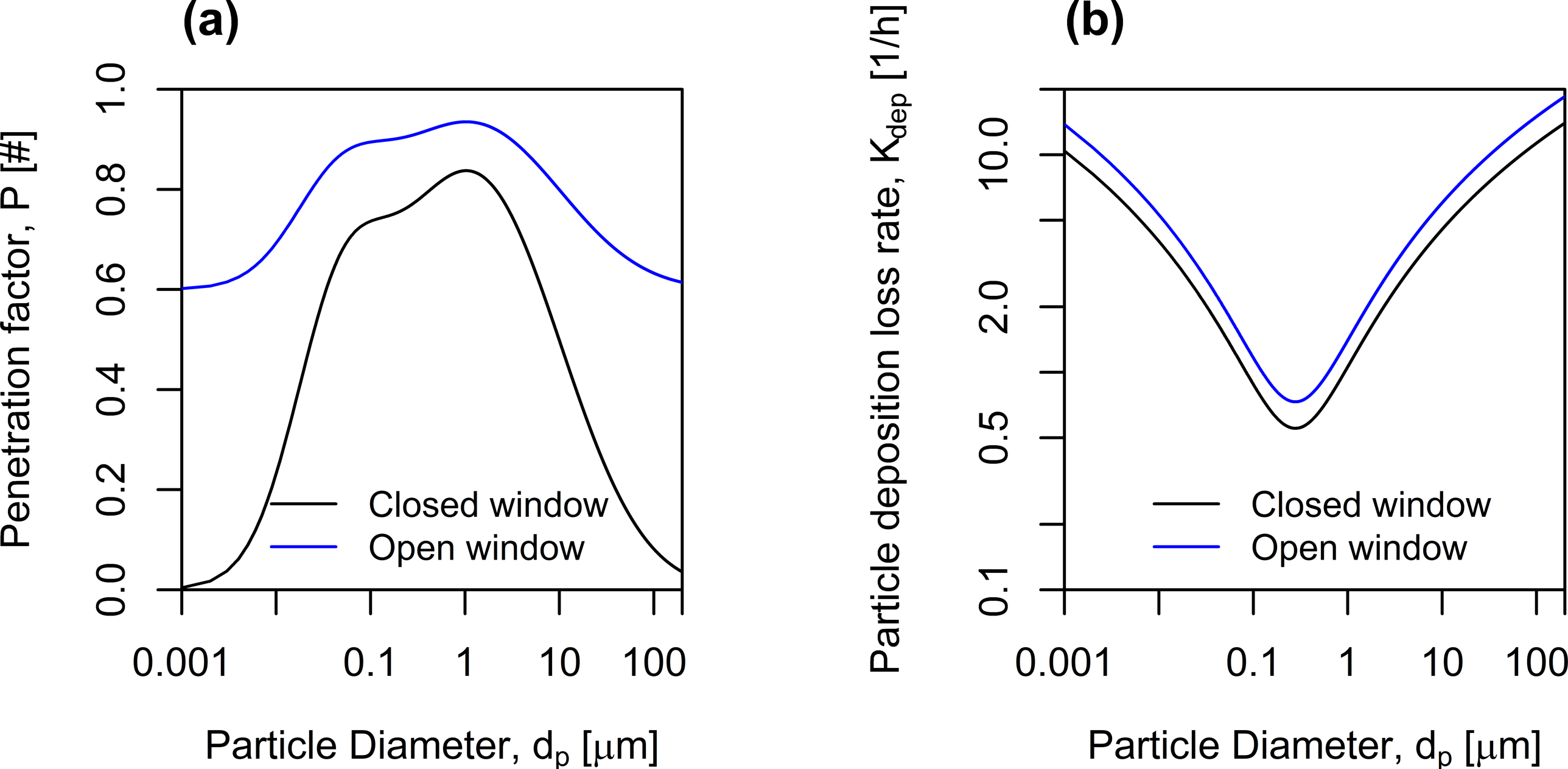


Figure A.1: (a) Size-resolved penetration factor, and (b) particle deposition loss rate in open and closed window scenario. The values for particles of size less than 10 *µm* were taken from El Orch et al[^10^.](#_bookmark48) The values were simply extrapolated for higher particle sizes.

The parameters of the tri-modal log-normal distribution obtained after the curve-fit to Figure 3 (a) from El Orch et al^10^ representing the penetration factor is given in [Table A.4.](#_bookmark6)

Table A.4: Penetration factor log-normal distribution parameters obtained by curve-fit to size-resolved penetration factor data in El Orch et al^10^.

|  | Mode 1 | | | Mode 2 | | | Mode 3 | | |
| --- | --- | --- | --- | --- | --- | --- | --- | --- | --- |
| Units | Total | Mean | Log10(SD) | Total | Mean | Log10(SD) | Total | Mean | Log10(SD) |
| (#) | 77.29 | 0.1388 | 0.4727 | 37360 | 76.97 | 0.8068 | 2716 | 7.996 | 0.7924 |

### Filtration efficiency and pressure drop data

The filtration efficiency (FE) data as reported in Hecker and Hofacre for the air cleaners selected in this study is given in [Figure A.2.](#_bookmark7)


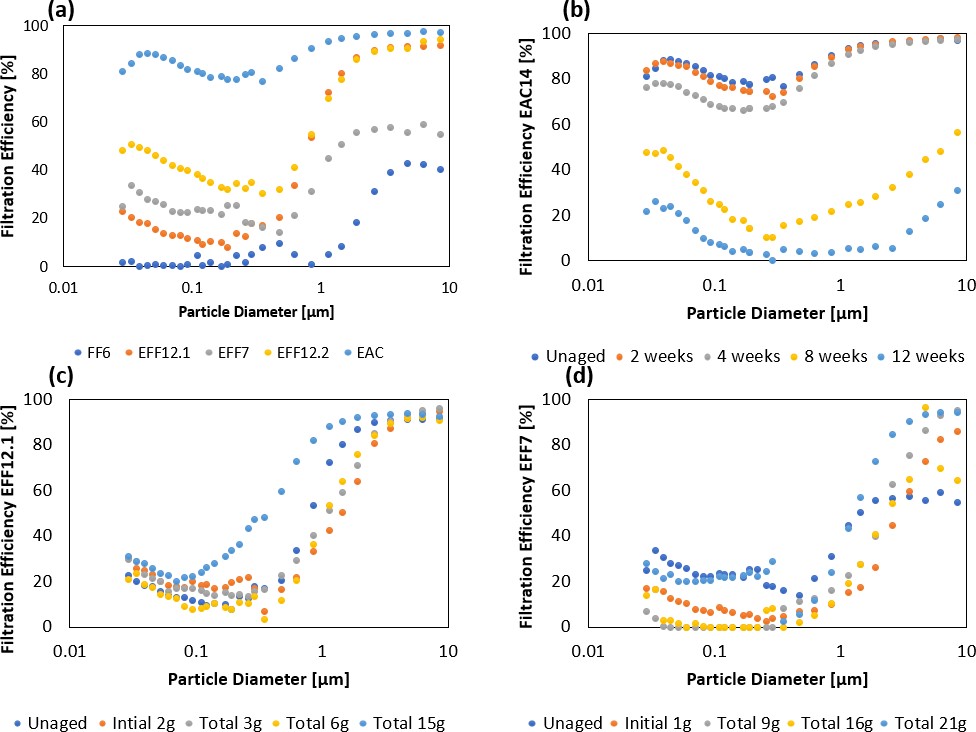


Figure A.2: The filtration efficiency (FE) data collected from Hecker and Hofacre for the selected air cleaners - (a) for all the new devices, (b) old EAC14, (c) old EEF12.1 and (d) old EEF7

The pressure drop data as reported in Hecker and Hofacre for the air cleaners selected for old device case in this study is given in [Table A.5.](#_bookmark8)

Table A.5: The pressure drop [Pa] data collected for old device case from the report by Hecker and Hofacre

|  | EAC14 | EFF12.1 | EFF7 |
| --- | --- | --- | --- |
| 2 weeks | 12.44 | 124.4 | 39.81 |
| 4 weeks | 12.44 | 144.4 | 57.23 |
| 8 weeks | 17.42 | 121.9 | 47.28 |
| 12 weeks | 14.93 | 435.5 | 64.70 |

### Curve-fit to obtain size-resolved filtration efficiency

The experimental FE data reported in Hecker and Hofacre[^1^](#_bookmark39) were for particles of diameter between 0.03 to 10 *µm*. Continuous functions were required as only 29 experimental values at discrete diameters were available. Also, there was a need to extrapolate the efficiencies beyond 10 *µm*. The mass deposited over the filter also comprised of PM of size above 10 *µm*. This was important for the aged device case.

The equations of single fiber filtration efficiency (SFFE) theory were used to obtain the full range of data required in this study for FFs and EFFs. In the case of EAC, it was not possible to use the common Deutsch equation for ESP efficiency estimation. This was because of the presence of pre-filter mesh and post-activated carbon filter. These also affected the FE. Thus, the empirical curve fit method, as suggested by Hecker and Hofacre[^1^](#_bookmark39) was used. A similar empirical curve fit could have been also used for FF and EFF. However, Hecker and Hofacre mentioned that their curve-fit method should not be used to extrapolate beyond 10 *µm*. Note that the scope of this study is limited to PM_2.5_ indoor concentration. Hence, FE for particles of size greater than 2.5 *µm* was only required if the FE of the aged device is considered to be dependent upon the mass accumulated over the filter, which consisted of entire PM. For EAC, deposited PM mass was not a problem as for the aging scenario, the FE is assumed to change only based on time of continuous usage. The detailed steps curve-fit can be found in [A.1.4.](#_bookmark9) The resulting curve-fit equations obtained for FE that were used to calculate indoor PM_2.5_ concentration can be found in [A.2.2.](#_bookmark24)

### Fibrous filters and Electret fibrous filters

The filtration efficiency equations from single fiber efficiency theory[^11^](#_bookmark49) is used for curve fitting to the experimental filtration efficiency of FF and EFF.

$$\eta=\left( 1-exp\left( -\frac{4\alpha\eta_{s}t}{\pi\left( 1-\alpha\right)\text{d}_{f}} \right) \right)*100$$

(A.5)

where, *η* is the size-resolved filtration efficiency of the filter [%]; *d_f_* represents the equivalent fiber diameter of filter; *α* is the filter solidity; *t* is the equivalent filter thickness; *η_s_* is the total single fiber efficiency given by [Equation A.6](#_bookmark10) [^11^;](#_bookmark49)

*η_s_* = 1 − (1 − *η_D_*) (1 − *η_R_*) (1 − *η_I_*) (1 − *η_DR_*) (1 − *η_E_*) (A.6)

Here, *η_D_*[^12^,](#_bookmark50) *η_R_*[^13^,](#_bookmark51) *η_DR_*[^13^,](#_bookmark51) *η_I_*[^13^,](#_bookmark51) and *η_E_*[^14^](#_bookmark52) are the single fiber efficiency due to diffusion, interception, interaction between diffusion and interception, inertial impaction, electrostatic attraction between charged fibers and uncharged particles collection mechanisms, respectively. These are given by [Equation A.7](#_bookmark11) - [A.11.](#_bookmark12)

$$\eta_{D}=0.84Pe^{-0.43}$$

(A.7)

$$\eta_{R}=\frac{\left( 1-\alpha\right)R^{2}}{Ku(1+R)}$$

(A.8)

$$\eta_{DR}=\frac{1.24R^{\frac{2}{3}}}{\left( Ku Pe \right)^{\frac{1}{2}}}$$

(A.9)

$$\eta_{I}=\frac{Stk^{3}}{Stk^{3}+0.77Stk^{2}+0.22}$$

(A.10)

$$\eta_{E}=\left( \frac{\left( 1-\alpha\right)}{Ku} \right)^{\frac{2}{5}}\left( \frac{\pi N_{DD}}{1+2\pi N_{DD}^{\frac{2}{3}}} \right)$$

(A.11)

where, *Pe* is the peclet number given by, $Pe=\frac{Ud_{f}}{D}$, where, $D=\frac{kC_{c}T}{3\pi{\mu d}_{p}}$ ,which is particle diffusion coefficient, *U* is the face velocity of filter, *C_c_* is the Cunningham slip corrections factor given by $C_{c}=1+\frac{\lambda}{d_{p}}\left( 2.34+1.05e^{- \frac{0.39d_{p}}{\lambda}} \right)$, *λ* is the air mean free path, *T* is the air temperature, *µ* is the dynamic viscosity of air, *d_p_* is the particle diameter; *R* is the interception parameter calculated as $R=\frac{d_{p}}{d_{f}}$; *Ku* is the Kuwabara flow field parameter given by *Ku* = −0*.*5 ln(*α*) − 0*.*75 + *α* − 0*.*25*α*^2^; *Stk* is the stokes number calculated as $Stk=\frac{\rho_{p}d_{p}^{2}C_{c}U}{18{\mu d}_{f}}$ where $\rho_{p}$ is the particle density; $N_{DD}$is given by $N_{DD}=\frac{2C_{C}Q^{2}d_{P}^{2}}{3\muƐ_{o}\left( 1+Ɛ_{f} \right)d_{f}U}\frac{\left( Ɛ_{P}-1 \right)}{{(Ɛ}_{P}+2)}$, where, *Q* represents the surface charge density on the fibers, *ε_P_* is the dielectric constant of particles, *ε_o_* is vacuum dielectric constant, *ε_f_* is the fiber dielectric constant.

Note that for uncharged fibrous filters, *η_E_* is zero.

The constants used during the curve fit is given in [Table A.6.](#_bookmark13) Note that the FE experiments were performed using the Potassium Chloride (KCl) particles. Thus, the *ρ_p_* and *ε**_p_* of KCl are used.

Table A.6: Constants used in the curve fit equations for FF and EFF

| Parameters | Values |
| --- | --- |
| Air dynamic viscosity, $\mu$ $\left[ \frac{kg}{ms} \right]$ | 0.00001834 |
| Particle density, $\rho_{p} \left[ \frac{kg}{m^{3}} \right]$ | 1984 |
| Boltzmann constant, $k\left[ \frac{J}{K} \right]$ | 1.38E-23 |
| Temperature, $T$ [$K$] | 293.15 |
| Permittivity of vacuum, $Ɛ_{o}\left[ \frac{A^{2}s^{4}}{m^{3}kg s} \right]$ | 8.85419E-12 |
| Particle dielectric constant, $Ɛ_{p}$ | 4.68 |
| Air mean free path, $\lambda$ [$\mu m$] | 0.066 |

The variable parameters that are obtained from curve-fitting to the empirical values are - *t* [*µm*], *d_f_* [*µm*], *U* [$\frac{m}{s}]$, for fibrous filters. EFF curve fitting involves two addition parameters - *Q* [$\frac{C}{m^{2}}$], and *ε_f_*. It is important to mention that all the filters have the same face velocity. However, these filters are pleated filters. In such a case, the velocity in face of different pleats is different. Thus, the face velocity is used as a varying parameter in the curve fit equations.

### Electronic air cleaners

[Equation A.12](#_bookmark14) shows the empirical curve fit equation used for obtaining size resolved FE of EAC.[^1^](#_bookmark39)

$$\eta_{EAC}= 100-{10}^{a+b*Log{10d}_{P}+c*\left( Log{10d}_{P} \right)^{2}+d*\left( Log{10d}_{P} \right)^{3}}$$

(A.12)

Thus, the parameters of curve fit equation of EAC are - a, b, c and d.

### Indoor PM_2.5_ concentration

The [Equation A.13](#_bookmark16) was used to obtain indoor PM_2.5_ concentration for unaged filters.

$$C_{PM2.5}=\sum_{x=1}^{2500} N_{in, x}\rho_{p, x}\frac{{\pi d}_{p, x}^{3}}{6}$$

(A.13)

where, *C_PM2.5_* is PM_2.5_ concentration in indoor air [*µg/m*^3^], *N_in,x_* is the indoor number concentration of particle of size x *nm*, *ρ_p,x_* is the density of particle size x and *d_p,x_* is the diameter of particle of size x *nm*. The particles are assumed to be spherical in shape and a constant unit density is assumed for all particle sizes. Equation 1 (main text) is substituted in [Equation A.13](#_bookmark16) for unaged filter case.

The total PM concentration in the residential building was calculated by the following equation:

$${PM}_{Tot}=\sum_{x=1}^{\infty} N_{in, x}\rho_{p, x}\frac{{\pi d}_{p, x}^{3}}{6}$$

(A.14)

To calculate the indoor PM_2.5_ concentration in case of old device, in the main text, the methodology specific to EEF7 and EEF12.1 was given. The generalized equations are as follows. The time taken to deposit a particular mass *z* [g] is given by [Equation A.15.](#_bookmark17)

$$tj\left( zj \right)=\frac{zj}{m_{f, j}Q_{r} *24*{10}^{-6}}$$

$$tl=90-\sum_{l=1}^{j-1} tj\left( zj \right)$$

(A.15)

The indoor number distribution for a particular efficiency curve was calculated as follows:

$$C_{in,\eta_{j-1}}=N_{o}\frac{P\text{λ}_{i}}{\text{λ}_{i}+\text{ }K_{dep}+\text{λ}_{R}\eta_{j-1}}+\sum_{k=1}^{n} \frac{\frac{E_{K}}{V}}{\text{λ}_{i}+\text{ }K_{dep}+\text{λ}_{R}\eta_{j-1}}$$

(A.16)

Finally, the average indoor PM_2.5_ concentration, over the time of 90 days was calculated as follows:

$$C_{PM2.5, avg}=\frac{\left( \left( \sum_{j=1}^{l-1} C_{PM2.5, \eta_{j-1}}*tj\left( zj \right) \right)+C_{PM2.5, \eta_{l-1}}tl \right)}{90}$$

(A.17)

The simple representation of the methodology for old device is given in [Figure A.3.](#_bookmark18)


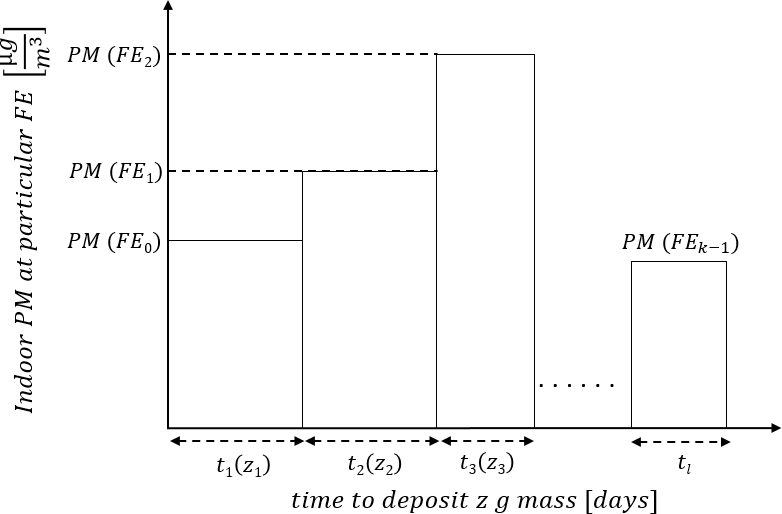
Figure A.3: Schematic to show the calculation of time weighted indoor PM_2.5_ concentration

Elaborated method for aged device case PM_2.5_ concentration- The data of EFF7 were available at subsequent loading of 1, 8, 7, and 5 g. The FE was assumed to be same as that of new device until 1 g is deposited, and is the same as unaged FE [(Figure A.2](#_bookmark7) (d) in [A.1.3).](#_bookmark2) The time to deposit 1 g was calculated by assuming the unaged FE. After accumulation of 1 g, it was assumed that FE changes to that shown for 1 g and remains the same until an additional 8 g is deposited on the filter. The time to deposit an additional 8 g was calculated using FE at 1g deposition. Similarly, after an additional 8 g deposition, the FE was assumed to change to that shown for 8 g deposition. The process was continued until the total time added up to 90 days. Finally, the indoor PM_2.5_ concentration was calculated at all the FE curves. The time-weighted average of indoor PM_2.5_ concentration was taken by taking the times calculated for depositing a particular loaded mass. The same methodology was followed to calculate average indoor PM_2.5_ concentration in the case of aged EFF12.1. However, in this case, the FE curves were available at the subsequent deposition of 2, 1, 3, and 9. The time to deposit these masses was first calculated. Afterward, the time-weighted average was taken. For more clarification of this method, A.1.5 can be referred to.

### Figure of merit (FOM)

FOM represents the ratio between the FE and PD. As the FE is particle size-dependent, so is the FOM. The FOM was calculated by [Equation A.18.](#_bookmark20)

$$FOM=-\frac{\ln\left( 1-\frac{\eta}{100} \right)}{{\Delta p}_{eff}}$$

where, *η* is the size resolved FE [%], and ∆*p_eff_* is the effective pressure drop [*Pa*] of the air cleaning device.

∆*p_eff_* can be considered a representative of power consumption. For EAC, additional power is required to charge the particles. The device power (*P_device_*) was converted to device pressure drop (∆*p_device_*) by ∆*p_device_* = *P_device_η_f_ /Q_r_*. Here, *η_f_* is the fan efficiency and *Q_r_* is the recirculation airflow rate [*m*^3^*/s*] across the air cleaning device. The effective pressure drop of EAC (*Pa*) is now calculated as the sum of ∆*p_device_* and ∆*p_f_* , i.e., ∆*p_eff_* = ∆*p_device_* + ∆*p_f_* . For EAC14, as the effective PD also depends upon the fan efficiency (*η_f_* ), the FOM was calculated for two values of *η_f_* , i.e., 0.5 and 1. Although, fan efficiency of 1 is almost impossible to achieve, it can provide the worst effective PD of EAC14.

The FOM can provide a ranking of air-cleaning devices for different particulate sizes. However, it cannot differentiate between the suitability of filters in different indoor environments ([A.1.6).](#_bookmark19)

### DALYs

Equation 3 (main article) is derived from a general formula for determining DALYs associated with indoor emissions [*DALY s/kgPM*_2_*_._*_5_*_emitted_*], i.e., *CF_PM_*_2_*_._*_5_*_emissions_*_→_*_DALY_* = *EF.IF* ; where *CF_PM_*_2_*_._*_5_*_emissions_*_→_*_DALY_* is the characterization factor to calculate DALYs due to PM_2.5_ emissions. IF is the PM_2.5_ intake fraction calculated as $IF=\frac{B.N}{V.Kex}$; where, B is the volume of air intake by one person in one year [*m*^3^*/yr/person*], N is the number of persons in the room, *K_ex_* is the overall ventilation rate in the indoor environment [*h*^−1^]. The indoor PM_2.5_ emissions divided by the *K_ex_*, *t_r_* and room’s volume *V* is the PM_2.5_ concentration in the room (Equation 1 in main text).

*EF_PM_*_2_*_._*_5_*_→DALY_* was taken as 78 to 110 *DALY/kgPM*_2_*_._*_5_*inhaled*, from the study by Gronlund et al[^15^.](#_bookmark53) Their study first calculated the population-weighted average of the concentration-response factors (CRF, annual mortality rate per *µg/m*^3^ PM_2.5_ inhaled) in 63 US Standard Metropolitan Statistical Areas (SMSAs) for mortality for different age groups (30-59, 60-69, and older than 70) and respective causes of death. CRF was then multiplied by the severity factors for the respective diseases. Overall EF was calculated as the product of CRF, IR, and severity factor for each age group, and the population- weighted mean was taken. The EF of 78 *DALY/kgPM*_2_*_._*_5_*inhaled* corresponds to only cardiopulmonary and lung cancer mortality results (plausible associations with PM_2.5_), and 110 *DALY/kgPM*_2_*_._*_5_*inhaled* is from all-cause results.

Tang et al[^16^](#_bookmark54) calculated that 1 kg CO_2_ emissions (customary 100-year global warming potential values) is associated with damage factor of 2 to 6.2×10^-7^ DALYs. Their study calculated the health damage factors corresponding to malaria, diarrhea, cardio- vascular disease, malnutrition, coastal flooding and inland flooding from CO_2_ emissions. This was done under four emission scenarios used by Intergovernmental Panel on Climate Change (IPCC) 2001 and 2007 report, namely, balanced emphasis on all energy sources (2×10^-7^ DALY/kg CO_2_), regionally oriented economic development (6.2×10^-7^ DALY/kg CO_2_), global environment sustainability (2.1×10^-7^ DALY/kg CO_2_), and local environmental sustainability (4.2×10^-7^ DALY/kg CO_2_). The overall range was directly used for *CF_CO_*_2→_*_DALY_* .

## Results and discussions

### Indoor PSD’s: No filter scenario

The indoor particle mass distribution obtained in no-filter case for all the scenarios is given in [Figure A.4.](#_bookmark22) It can be seen that PM mass from indoor emission sources mainly comprise of particles of size less than 10 *µm*. However, visible indoor particles mass of size above 10 *µm* can be seen for particles from outdoor rural and urban origin. One important message that the figure gives is that the mass distribution is dominated by particles of size lesser than 1 *µm*, which are considered to be more toxic as they can penetrate deeper into the lungs. The curves for particles of outdoor origin shows slightly lower concentration for all particles sizes in case of closed window scenario compared to the open window scenario. On the other hand, the curves for indoor emission sources shows higher concentration in closed window scenario compared to open window situation. This shows the plausibility of the infiltration and dilution factors used in this study. The indoor particle number distribution is given in [Figure A.5.](#_bookmark23)


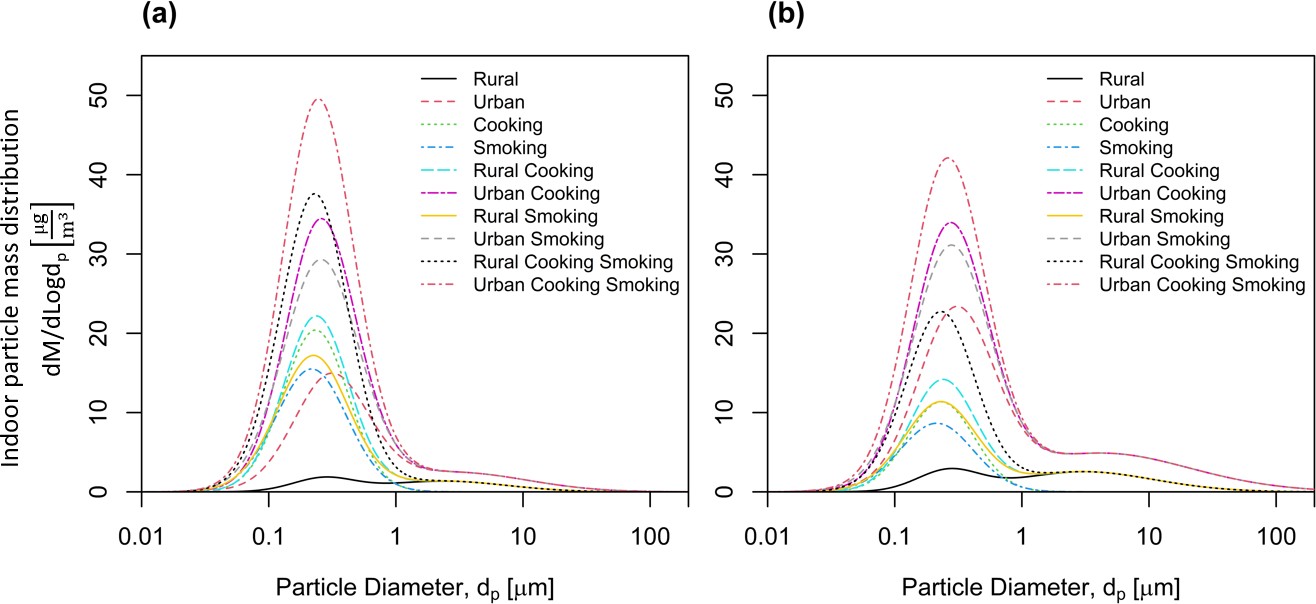
Figure A.4: Indoor PSD mass concentration in case of no filter


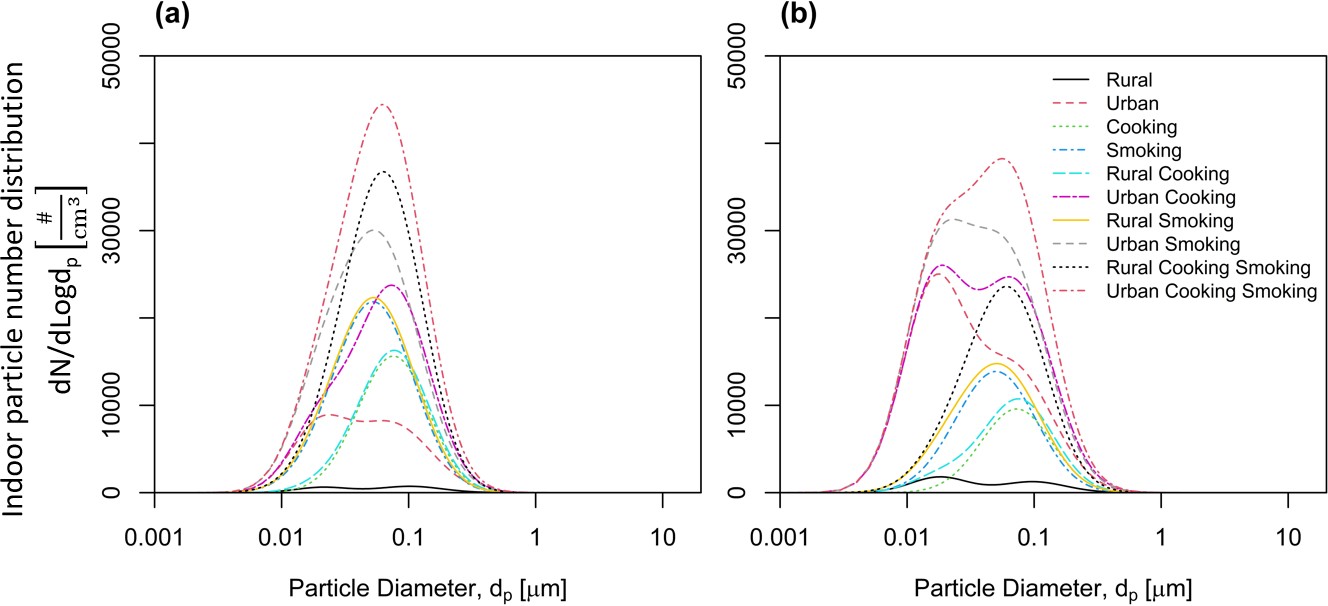


Figure A.5: Indoor PSD number concentration in case of no filter

### Curve-fit filtration efficiency

The size resolved FE obtained from curve fitting is shown in [Figure A.6.](#_bookmark25) For all new filters and aging case of EAC14, the curves are only shown for particles of size less than 2.5 *µm*. Excellent fit against the experimental values reported by Hecker and Hofacre can be seen for particles of size above 0.05 *µm*. Below this, slight overestimation in FE by the obtained curve-fits can be seen. It can be seen that even the highest indoor mass below this size is very low. Thus, it can be said that the error from this overestimation is minimal. In case of EFF7 and EFF12 with aging, overestimation in FE can be observed for particles of size greater than 2.5 *µm*. The impact of this can result in faster loading compared to reality.


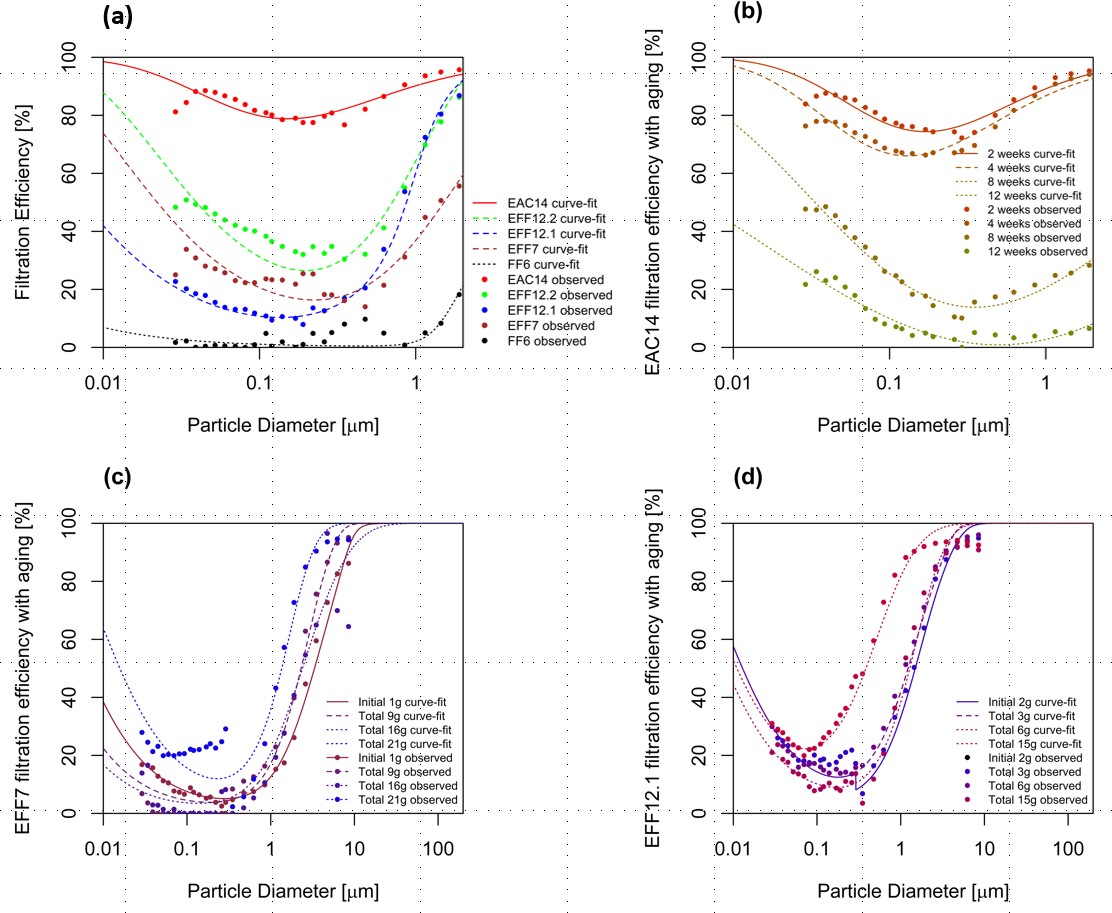


Figure A.6: Curve-fits comparison to FE data collected - (a) all new air cleaners; (b) EAC14; (c) EFF7; (d) EFF12.1

The parameters of the curve-fit can be found in [Table A.7,](#_bookmark26) [A.8,](#_bookmark27) [A.9](#_bookmark28) and [A.10.](#_bookmark29) Note that the values of these parameters should not matter as far as they provide plausible shape of particle size-resolved FE curves.

Table A.7: Curve fit parameters of new devices (EFF12.1 and FF6)

|  | EFF12.2 | FF6 |
| --- | --- | --- |
| df [µm] | 9 | 15 |
| Solidity | 0.05 | 0.05 |
| t [µm] | 900 | 142.53 |
| U [m/s] | 0.075 | 0.389 |
| Q [µC/m2] | 25 |  |
| *ϵ_f_* | 5 |  |
| RSQ | 0.97 | 0.95 |

Table A.8: Curve fit parameters of new and old EAC14

| EAC14 | New | 2 weeks | 4 weeks | 8 weeks | 16 weeks |
| --- | --- | --- | --- | --- | --- |
| a | 0.992 | 1.0353 | 1.1219 | 1.89963 | 1.987953 |
| b | -0.7057 | -0.8744 | -0.7987 | -0.15065 | -0.05478 |
| c | -0.2291 | -0.3793 | -0.2345 | -0.14356 | -0.092346 |
| d | 0.1645 | 0.1645 | 0.1645 | 0.03404 | -0.003976 |
| RSQ | 0.83 | 0.92 | 0.95 | 0.97 | 0.92 |

Table A.9: Curve fit parameters of new and old EEF12.1

| EEF12.1 | New | 1 g | Additional 8 g | Additional 7 g | Additional 5 g |
| --- | --- | --- | --- | --- | --- |
| df [µm] | 19.7 | 90 | 90 | 949 | 137 |
| Solidity | 0.065 | 0.11 | 0.66 | 0.65 | 0.38 |
| t [µm] | 613 | 2620 | 100 | 404 | 1750 |
| U [m/s] | 0.01 | 0.071 | 0.107 | 0.002 | 0.054 |
| Q [µC/m2] | 15.7 | 26.3 | 26.3 | 26.3 | 26.3 |
| *ϵ_f_* | 5 | 5 | 5 | 5 | 5 |
| RSQ | 0.8 | 0.99 | 0.99 | 0.92 | 0.94 |

Table A.10: Curve fit parameters of new and old EEF7

| EEF7 | New | 2 g | Additional 1 g | Additional 3 g | Additional 9 g |
| --- | --- | --- | --- | --- | --- |
| df [µm] | 3.44 | 55.3 | 31 | 105 | 192 |
| Solidity | 0.05 | 0.52 | 0.07 | 0.39 | 0.54 |
| t [µm] | 100 | 100 | 1710 | 827 | 232 |
| U [m/s] | 0.219 | 0.092 | 0.092 | 0.092 | 0.002 |
| Q [µC/m2] | 33.8 | 33.8 | 33.8 | 33.8 | 33.8 |
| *ϵ_f_* | 3.93 | 3.93 | 3.93 | 3.93 | 3.93 |
| RSQ | 0.99 | 0.98 | 0.99 | 0.98 | 0.99 |

### Indoor PM_2.5_ concentration


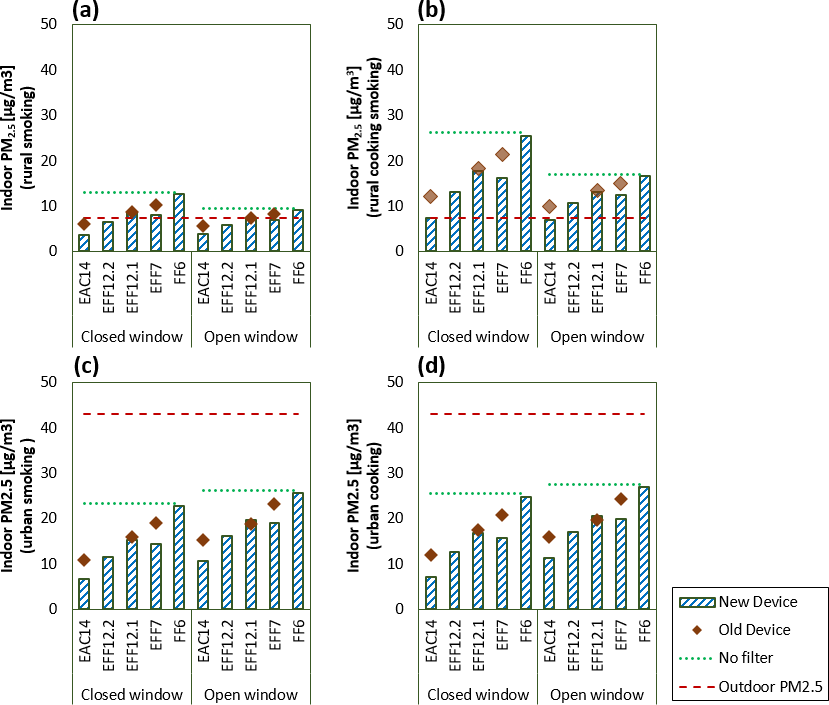


Figure A.7: Comparison of different air cleaning technologies in terms of indoor PM_2.5_ concentration with constant density assumption in - (a) Rural, (b) Rural cooking, (c) Rural cooking smoking, (d) Urban, (e) Urban cooking smoking scenario. Lower concentration implies better performance

The time metric for EFF7 is given in [Table A.11.](#_bookmark31)

Table A.11: Time metric for EFF7 (constant unit density assumption)

|  | Closed window | | | | | Open window | | | | |
| --- | --- | --- | --- | --- | --- | --- | --- | --- | --- | --- |
|  | Initial 1g | Total 9g | Total 16g | Total 21g | More than 21g | Initial 1g | Total 9g | Total 16g | Total 21g | More than 21g |
| Rural | 42.66 | 47.34 | 0.00 | 0.00 | 0.00 | 16.53 | 73.47 | 0.00 | 0.00 | 0.00 |
| Urban | 11.21 | 78.79 | 0.00 | 0.00 | 0.00 | 4.45 | 52.22 | 33.33 | 0.00 | 0.00 |
| Cooking | 19.07 | 70.93 | 0.00 | 0.00 | 0.00 | 27.92 | 62.08 | 0.00 | 0.00 | 0.00 |
| Smoking | 22.16 | 67.84 | 0.00 | 0.00 | 0.00 | 32.25 | 57.75 | 0.00 | 0.00 | 0.00 |
| Rural Cooking | 13.18 | 76.82 | 0.00 | 0.00 | 0.00 | 10.38 | 79.62 | 0.00 | 0.00 | 0.00 |
| Urban Cooking | 7.06 | 82.94 | 0.00 | 0.00 | 0.00 | 3.84 | 47.81 | 38.35 | 0.00 | 0.00 |
| Rural Smoking | 14.58 | 75.42 | 0.00 | 0.00 | 0.00 | 10.93 | 79.07 | 0.00 | 0.00 | 0.00 |
| Urban Smoking | 7.44 | 82.56 | 0.00 | 0.00 | 0.00 | 3.91 | 48.29 | 37.79 | 0.00 | 0.00 |
| Rural Cooking Smoking | 8.26 | 81.74 | 0.00 | 0.00 | 0.00 | 7.85 | 82.15 | 0.00 | 0.00 | 0.00 |
| Urban Cooking Smoking | 5.35 | 80.66 | 3.98 | 0.00 | 0.00 | 3.43 | 44.50 | 38.32 | 3.75 | 0.00 |

The time metric for EFF12.1 is given in the following table:

Table A.12: The time metric for EFF12.1 (constant unit density assumption)

|  | Closed Window | | | | |  | Open Window | | | |
| --- | --- | --- | --- | --- | --- | --- | --- | --- | --- | --- |
|  | Initial 2g | Total 3g | Total 6g | Total 15g | More than 15g | Initial 2g | Total 3g | Total 6g | Total 15g | More than 15g |
| Rural | 80.08 | 9.92 | 0.00 | 0.00 | 0.00 | 30.73 | 16.84 | 42.43 | 0.00 | 0.00 |
| Urban | 22.32 | 12.38 | 34.26 | 21.04 | 0.00 | 8.76 | 4.84 | 13.51 | 43.31 | 19.58 |
| Cooking | 45.92 | 24.16 | 19.92 | 0.00 | 0.00 | 68.57 | 21.43 | 0.00 | 0.00 | 0.00 |
| Smoking | 54.52 | 28.00 | 7.48 | 0.00 | 0.00 | 81.16 | 8.84 | 0.00 | 0.00 | 0.00 |
| Rural Cooking | 29.18 | 15.59 | 42.93 | 2.30 | 0.00 | 21.22 | 11.55 | 32.24 | 25.00 | 0.00 |
| Urban Cooking | 15.02 | 8.18 | 22.48 | 44.32 | 0.00 | 7.77 | 4.27 | 11.87 | 38.75 | 27.34 |
| Rural Smoking | 32.44 | 17.10 | 40.47 | 0.00 | 0.00 | 22.29 | 12.04 | 33.84 | 21.82 | 0.00 |
| Urban Smoking | 15.84 | 8.58 | 23.68 | 41.90 | 0.00 | 7.91 | 4.34 | 12.08 | 39.35 | 26.32 |
| Rural Cooking Smoking | 19.01 | 10.01 | 27.52 | 33.46 | 0.00 | 16.82 | 9.07 | 25.13 | 38.98 | 0.00 |
| Urban Cooking Smoking | 11.77 | 6.33 | 17.38 | 54.51 | 0.00 | 7.09 | 3.88 | 10.75 | 35.55 | 32.73 |

The time metric for particle size-dependent density assumption showing the time required to accumulate a particular mass over aged EFF7 and EFF12.1 is given in [Table A.13](#_bookmark33) and [Table A.14,](#_bookmark34) respectively in [A.2.3.](#_bookmark30) Compared to the unit density assumption, less time was required to accumulate the same mass. However, this still does not change the ranking for aged devices. Comparing the new and aged device case results, the overall effectiveness of the aged EAC is lower than that of the new EAC. This was expected as the FE reduced with time [(Figure A.6](#_bookmark25) (b) in [A.2.2).](#_bookmark24) For EFF7 and EFF12.1, the summary of time required to load a particular amount of mass over the filter is given in [Table A.11](#_bookmark31) and [A.12,](#_bookmark32) respectively in [A.2.3.](#_bookmark30) For EFF7, it was seen that in 90 days, in no scenario, more than 21g mass was deposited over the filter. The FE of EFF7 increased significantly only after accumulation of 21g [(Figure A.6](#_bookmark25) (c) in [A.2.2).](#_bookmark24) Hence, the PM_2.5_ indoor concentration is higher in all the scenarios when aging is considered compared to the new device case. On the contrary, in the case of EFF12.1, for open window situation combined with scenarios having urban location, 15g was deposited at least 19 days before the filter end of life (90 days). After 15g deposition, the FE of EFF12.1 increased significantly [(Figure A.6](#_bookmark25) (d) in [A.2.2).](#_bookmark24) Thus, for these scenarios, aged device performance is better compared to the new filter. Even though the PM_2.5_ concentration is higher in the closed window case, faster loading is achieved in the open window scenario. This is because the loading depends upon total PM mass and not only PM_2.5_ concentration. It can be seen in [Figure A.4](#_bookmark22) (in [A.2.1)](#_bookmark21) that indoor PM mass concentration for particles of diameter above 2.5 *µm* is higher in open window compared to closed window scenario. The FE of EFF’s initially decreases with usage due to the shielding of filter charge by the accumulated dust particles. However, after some accumulation, the FE starts increasing similar to FF’s [[1].](#_bookmark39) Thus, it can be said the faster the mass accumulates over a filter, the better overall performance can be achieved over 3 months.


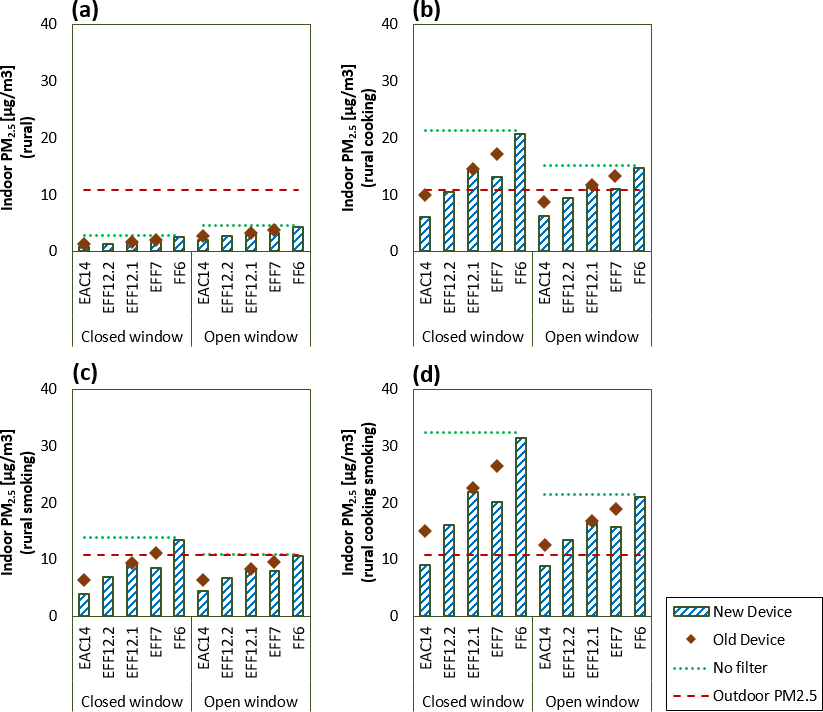


Figure A.8: Indoor PM_2.5_ concentration when the unit density assumption was changed to that given in Sec. 2.3 for residences located in rural area


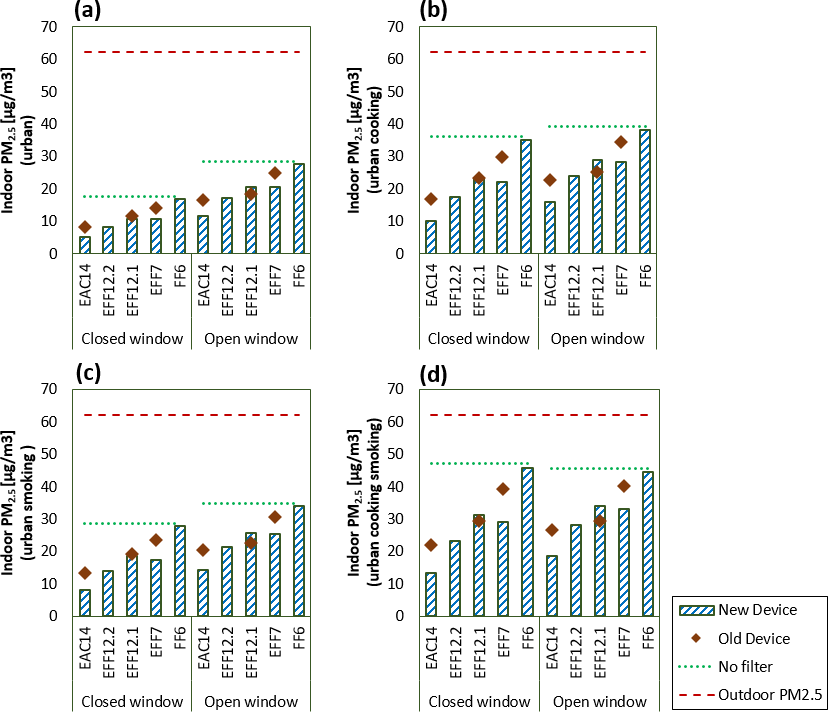


Figure A.9: Indoor PM_2.5_ concentration when the unit density assumption was changed to that given in Sec. 2.3 for residences located in urban area

Table A.13: Time metric EEF7 (particle size-dependent density assumption)

| Scenario | Closed window | | | | | Open window | | | | |
| --- | --- | --- | --- | --- | --- | --- | --- | --- | --- | --- |
|  | Initial 1g | Total 9g | Total 16g | Total 21g | More than 21g | Initial 1g | Total 9g | Total 16g | Total 21g | More than 21g |
| Rural | 24.71 | 65.29 | 0.00 | 0.00 | 0.00 | 9.03 | 80.97 | 0.00 | 0.00 | 0.00 |
| Urban | 6.78 | 80.54 | 2.68 | 0.00 | 0.00 | 2.48 | 26.85 | 22.42 | 16.10 | 22.15 |
| Cooking | 13.66 | 76.34 | 0.00 | 0.00 | 0.00 | 19.99 | 70.01 | 0.00 | 0.00 | 0.00 |
| Smoking | 22.16 | 67.84 | 0.00 | 0.00 | 0.00 | 32.25 | 57.75 | 0.00 | 0.00 | 0.00 |
| Rural Cooking | 8.80 | 81.20 | 0.00 | 0.00 | 0.00 | 6.22 | 73.97 | 9.81 | 0.00 | 0.00 |
| Urban Cooking | 4.53 | 60.98 | 24.49 | 0.00 | 0.00 | 2.21 | 25.19 | 21.31 | 15.25 | 26.05 |
| Rural Smoking | 11.68 | 78.32 | 0.00 | 0.00 | 0.00 | 7.06 | 79.27 | 3.67 | 0.00 | 0.00 |
| Urban Smoking | 5.19 | 67.06 | 17.75 | 0.00 | 0.00 | 2.30 | 25.77 | 21.73 | 15.58 | 24.62 |
| Rural Cooking Smoking | 6.30 | 83.70 | 0.00 | 0.00 | 0.00 | 5.22 | 66.34 | 18.45 | 0.00 | 0.00 |
| Urban Cooking Smoking | 3.76 | 52.92 | 33.32 | 0.00 | 0.00 | 2.07 | 24.24 | 20.68 | 14.79 | 28.23 |

Table A.14: Time metric EEF12.1 (particle size-dependent density assumption)

| Scenario | Closed window | | | | | Open window | | | | |
| --- | --- | --- | --- | --- | --- | --- | --- | --- | --- | --- |
|  | Initial 2g | Total 3g | Total 6g | Total 15g | More than 15g | Initial 2g | Total 3g | Total 6g | Total 15g | More than 15g |
| Rural | 46.41 | 25.22 | 18.36 | 0.00 | 0.00 | 16.89 | 9.14 | 26.33 | 37.65 | 0.00 |
| Urban | 13.39 | 7.37 | 20.58 | 48.67 | 0.00 | 4.86 | 2.64 | 7.48 | 23.61 | 51.42 |
| Cooking | 32.60 | 17.29 | 40.11 | 0.00 | 0.00 | 48.59 | 26.26 | 15.15 | 0.00 | 0.00 |
| Smoking | 54.52 | 28.00 | 7.48 | 0.00 | 0.00 | 81.16 | 8.84 | 0.00 | 0.00 | 0.00 |
| Rural Cooking | 19.15 | 10.26 | 28.35 | 32.25 | 0.00 | 12.53 | 6.78 | 19.10 | 51.59 | 0.00 |
| Urban Cooking | 9.49 | 5.16 | 14.28 | 47.56 | 13.50 | 4.41 | 2.40 | 6.75 | 21.65 | 54.78 |
| Rural Smoking | 25.07 | 13.27 | 37.20 | 14.46 | 0.00 | 13.98 | 7.51 | 21.39 | 47.12 | 0.00 |
| Urban Smoking | 10.75 | 5.83 | 16.22 | 53.44 | 3.76 | 4.58 | 2.49 | 7.02 | 22.38 | 53.53 |
| Rural Cooking Smoking | 14.17 | 7.51 | 20.69 | 47.63 | 0.00 | 10.86 | 5.84 | 16.36 | 53.90 | 3.04 |
| Urban Cooking Smoking | 8.08 | 4.36 | 12.04 | 40.70 | 24.82 | 4.19 | 2.27 | 6.37 | 20.62 | 56.55 |

###

### Power consumption

The power consumption when the particle size-dependent density was used is given in [Figure A.10](#_bookmark36).


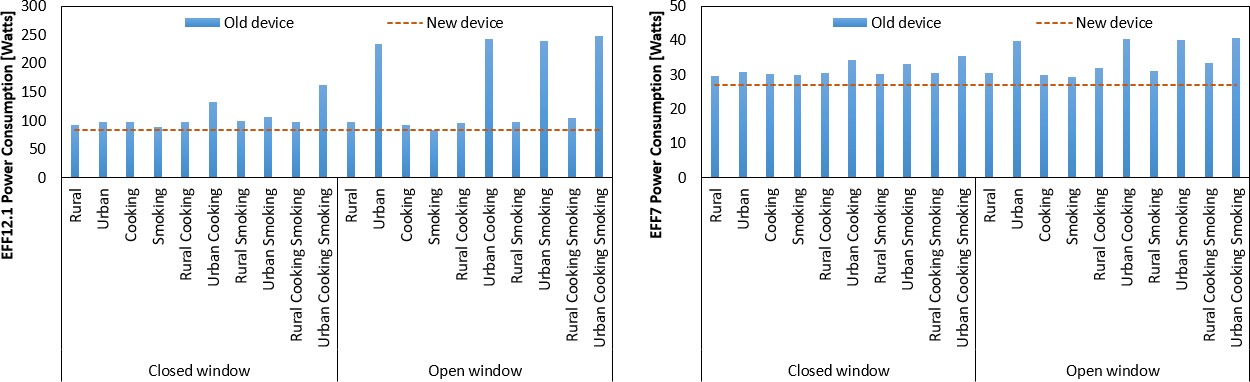


Figure A.10: Power consumption of old EEF12.1 and EEf7 when the unit density assump- tion was changed to that given in Sec. 2.3

### Figure of Merit

The FOM of all the new air cleaners is shown in [Figure A.11.](#_bookmark35) For all particle diameters between 0.01 to 2.5 *µm*, the FOM of FF6 is the least. Note that during the selection of air cleaners, FF6 (original name “NS”) was selected with a PD of 47 *Pa*. There was another FF named ”PP” reported by Hecker and Hofacre[^1^](#_bookmark39) (T[able A.2](#_bookmark1) in [A.1.2).](#_bookmark0) The PD of FF ”PP” is 45 *Pa* that is only 4.3% lower compared to that of FF6. It is shown in [Figure A.12](#_bookmark38) in [A.2.5](#_bookmark37) that after replacing the PD to 45 Pa for FF, the trend remains the same, which was expected. Thus, a generalized statement can be made that amongst the popular air cleaners in U.S. residences, the FF has the lowest FOM.


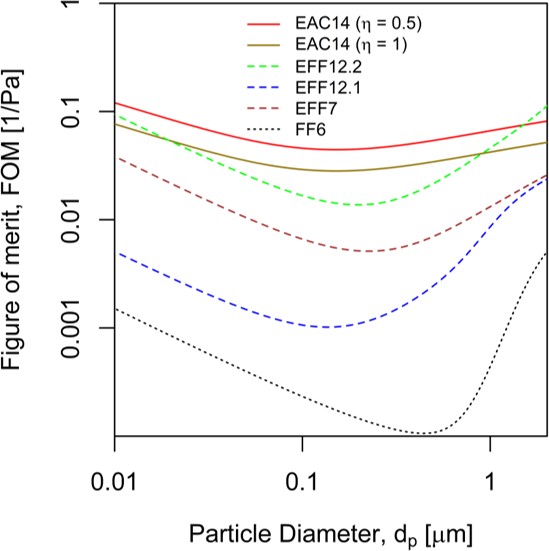


Figure A.11: Particle size-resolved figure of merit (FOM) of new air cleaners for PM size between 0.01 to 2.5 *µm*

Comparing EFFs to EAC, it can be seen that the EAC14 has the highest FOM, except for the particles of sizes above 1 *µm* when the *η_f_* is taken as 0.5. Even the worst-case FOM of EAC14 is the highest for the majority of the particle sizes (when *η_f_* = 1). Again, there were 3 EACs reported by Hecker and Hofacre (T[able A.2](#_bookmark1) in [A.1.2).](#_bookmark0) The [Figure A.12](#_bookmark38) in [A.2.5](#_bookmark37) shows that with the realistic *η_f_* of 0.5, the Unit A with worst PD amongst the 3 EACs, still has the highest FOM for the majority of the particle sizes. Thus, a generalized statement can be made that EAC’s have the highest FOM, followed by EFF’s with FF’s having the least FOM, for the majority of the particle sizes. This is only valid for the new device case. One limitation of the FOM metric is that it cannot be used for aged device scenario in its current format. On the other hand, the advantage is that the metric is robust. It is not affected by varying assumptions (except for value of *η_f_* ). This is not the case with the DALY metric developed in the current study.


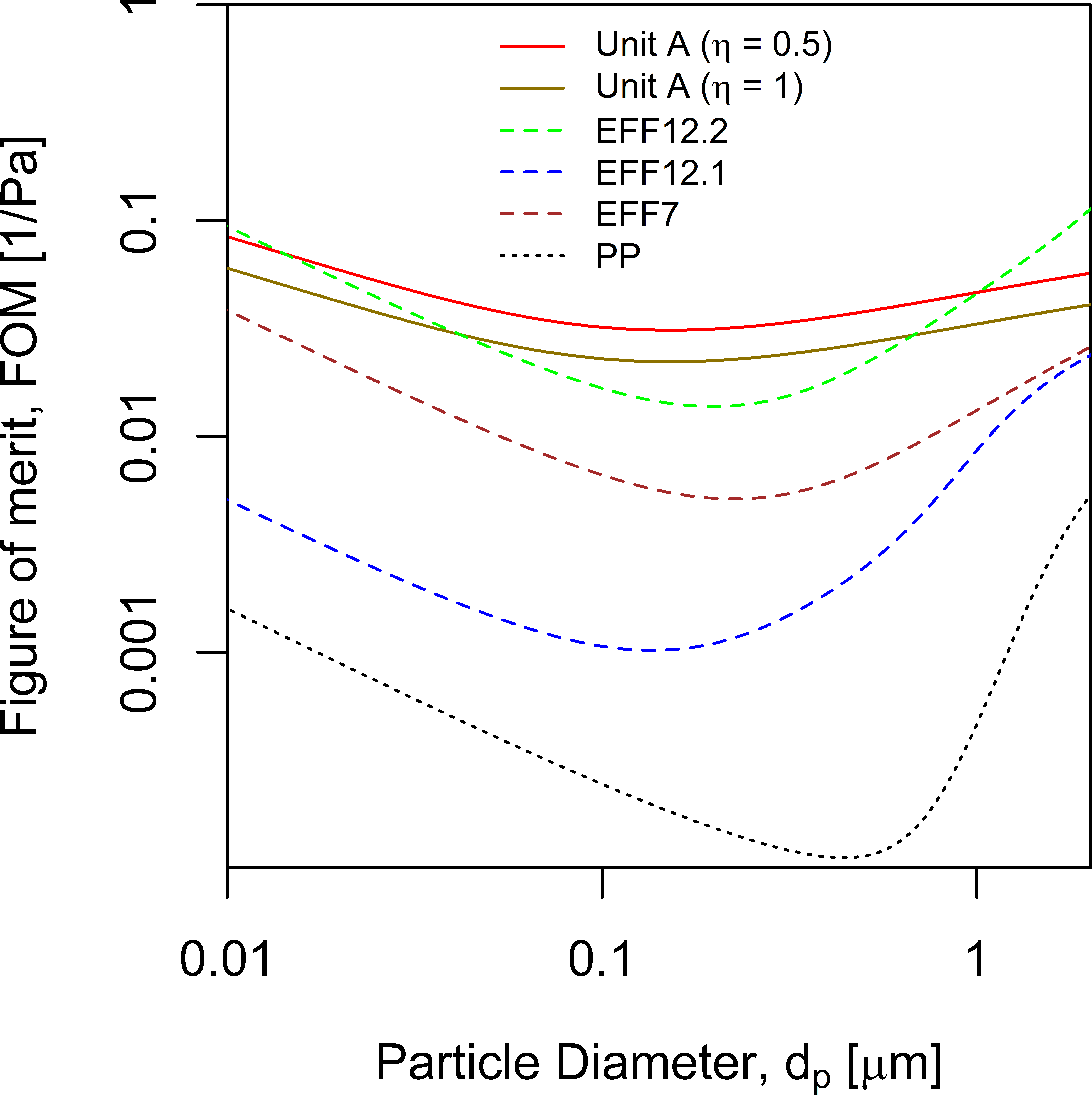


Figure A.12: Size-resolved figure of merit (FOM) of new air cleaners for PM size between 0.01 to 2.5 *µm*. Instead of PD of FF6, PD of PP is taken. Also, in EAC, Unit A is taken, which has the highest PD.

# References

- - 1. Hecker R, Hofacre KC. Development of performance data for common building air cleaning devices (final report no. EPA/600/R-08/013). US Environmental Protection Agency, Office of Research and Development/National Homeland Security Research Center Research Triangle Park, NC. 2008.
    2. Ruprecht Jaenicke. Tropospheric Aerosols. San Diego Academic Press. 1993; 1– 31.
    3. Riley WJ, McKone TE, Lai AC, Nazaroff WW. Indoor particulate matter of outdoor origin: importance of size-dependent removal mechanisms. Environmental science & technology. 2002; 36(2):200-7. issn: 0013936X. doi: [10.1021/es010723y](https://doi.org/10.1021/es010723y).
    4. Azimi P, Zhao D, Stephens B. Estimates of HVAC filtration efficiency for fine and ultrafine particles of outdoor origin. Atmospheric Environment. 2014; 98:337-46. doi: [10.1016/j.atmosenv.2014.09.007](https://doi.org/10.1016/j.atmosenv.2014.09.007).
    5. *Particulate Matter (PM2.5) Trends — US EPA*. url: [https://www.epa.gov/air-](https://www.epa.gov/air-trends/particulate-matter-pm25-trends) [trends/particulate-matter-pm25-trends](https://www.epa.gov/air-trends/particulate-matter-pm25-trends).
    6. Wallace LA, Emmerich SJ, Howard-Reed C. Source strengths of ultrafine and fine particles due to cooking with a gas stove. Environmental Science & Technology. 2004; 38(8):2304-11. issn: 0013936X. doi: [10.1021/es0306260](https://doi.org/10.1021/es0306260).
    7. Waring MS, Siegel JA. Particle loading rates for HVAC filters, heat exchangers, and ducts. Indoor air. 2008; 18(3):209-24. issn: 09056947. doi: [10.1111/j.1600-0668.2008.00518.x](https://doi.org/10.1111/j.1600-0668.2008.00518.x).
    8. Nazaroff WW, Klepeis NE. Environmental tobacco smoke particles. Indoor environment: airborne particles and settled dust. WILEY-VCH Verlag GmbH & Co. KGaA. 2003; 245-74. ISBN: 978-3-527-60920-8.
    9. Press Release. Smoking is down, but almost 38 million American adults still smoke. 2018.
    10. El Orch Z, Stephens B, Waring MS. Predictions and determinants of size-resolved particle infiltration factors in single-family homes in the US. Building and Environment. 2014; 74:106-18. issn: 03601323. doi: [10.1016/j.buildenv.2014.01.006](https://doi.org/10.1016/j.buildenv.2014.01.006).
    11. William C. Hinds. Aerosol Technology: Properties, Behavior, and Measurement of Airborne Particles. Wiley-Interscience. 1999; 182–205. isbn: 9781119130536.
    12. J. Wang, D. R. Chen, and D. Y.H. Pui. Modeling of filtration efficiency of nanopar- ticles in standard filter media. Journal of Nanoparticle Research 9.1. 2007; 109–115. issn: 13880764. doi: [10.1007/s11051-006-9155-9](https://doi.org/10.1007/s11051-006-9155-9).
    13. K. W. Lee and B. Y.H. Liu. Theoretical study of aerosol filtration by fibrous filters. Aerosol Science and Technology 1.2. 1982; 147–161. issn: 15217388. doi:

[10.1080/02786828208958584](https://doi.org/10.1080/02786828208958584).

- - 1. Zhuangbo Feng and Shi Jie Cao. A newly developed electrostatic enhanced pleated air filters towards the improvement of energy and filtration efficiency. Sustainable Cities and Society 49. 2019; 101569. issn: 22106707. doi: [10.1016/](https://doi.org/10.1016/j.scs.2019.101569) [j.scs.2019.101569](https://doi.org/10.1016/j.scs.2019.101569). url: <https://doi.org/10.1016/j.scs.2019.101569>.
    2. Gronlund CJ, Humbert S, Shaked S, O’Neill MS, Jolliet O. Characterizing the burden of disease of particulate matter for life cycle impact assessment. Air Quality, Atmosphere & Health. 2015; 8(1):29-46. issn: 18739326. doi: [10.1007/s11869-014-0283-6](https://doi.org/10.1007/s11869-014-0283-6).
    3. Tang L, Ii R, Tokimatsu K, Itsubo N. Development of human health damage factors related to CO2 emissions by considering future socioeconomic scenarios. The International Journal of Life Cycle Assessment. 2018; 23(12):2288-99. issn: 16147502. doi: [10.1007/s11367-015-0965-9](https://doi.org/10.1007/s11367-015-0965-9).
